# Bioinspired Morphology‐Decoupled Soft Gripper with Enhanced Bidirectional Grasping Capability

**DOI:** 10.1002/advs.75073

**Published:** 2026-04-02

**Authors:** Yedong Huang, Dazhong Yu, Baijin Mao, Fangming Li, Juntian Qu

**Affiliations:** ^1^ Shenzhen International Graduate School Tsinghua University Shenzhen China; ^2^ Ocean Decade International Cooperation Center Qingdao China; ^3^ Fujian Ocean Innovation Center Xiamen China; ^4^ School of Mechatronical Engineering Beijing Institute of Technology Beijing China; ^5^ State Key Laboratory of Explosion Science and Safety Protection School of Mechatronical Engineering Beijing Institute of Technology Beijing China

**Keywords:** grippers and other end‐effectors, soft gripper, soft robot materials and design, soft robot

## Abstract

The integrated handling of dynamic and static targets remains a formidable challenge for soft grippers. Although cross‐modal grippers combine active and passive modes, they are constrained by inherent conflicts among precision, speed and strength, leading to bidirectional performance degradation. Here, inspired by principles of predation, we propose a strategy to decouple cross‐modal grasping via dual morphological configurations. Guided by this strategy, we develop rigid‐soft fingers and metamaterial palms. Through coordinated morphological synergy, we achieve optimization and fractal utilization of different grasping mechanisms. In the parallel configuration, the gripper employs an active compliant contact grasping mechanism. The cage configuration employs a soft constraint mechanism for the passive capture paradigm, combining spatial confinement and energy dissipation. Experiments demonstrate enhanced bidirectional grasping of static and dynamic targets, including multi‐object storage and transfer, and uncooperative targets capture. This work achieves system‐level optimization of grasping modes and multivariate abilities, opening new avenues for soft gripper paradigms.

## Graphical abstract

Inspired by biological predation, a novel gripper decouples cross‐modal grasping via dual morphological configurations. Synergistically integrating hybrid rigid and soft coupled fingers with a metamaterial palm, the system performs active compliant grasping for static objects and passive cage capture for dynamic targets. This approach overcomes inherent performance conflicts, achieving remarkable system‐level optimization for versatile robotic manipulation.

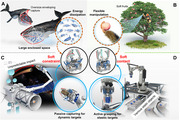

## 1 Introduction

Grasping represents one of the fundamental capabilities for robots to perform complex tasks. Compared to conventional rigid grippers, soft grippers have garnered significant interest for their transformative advancement [1]. Due to their inherent adaptability and continuously compliant deformation, making them a prominent research focus for compliant manipulation, human‐robot interaction, and unstructured environments [2]. At its core, their transformative significance lies in establishing a new paradigm of physical interaction. This shift enables non‐adversarial, universal soft contact and more physically intelligent adaptive force control, thereby superseding the paradigm of precise control and response models. However, the intrinsically low modulus of soft materials inherently limits their force transmission efficiency. Consequently, soft grippers are unable to sustain their advantages during rapid and violent interactions with highly dynamic targets [3, 4].

Current research mainly focuses on amplifying the intrinsic advantages of single‐mode soft grippers in specific scenarios. On the one hand, material [5, 6, 7, 8, 9, 10, 11] and structural [12, 13, 14, 15, 16, 17, 18, 19] optimizations enable active soft hands to achieve more dexterous and compliant grasping of complex static targets [1, 2, 5, 6, 7, 8, 9, 10, 11, 12, 13, 14, 15, 16, 17, 18, 19]. On the other hand, various designs of bistable and other instability structures provide passive triggering for high‐speed gripping of dynamic targets. [20, 21, 22, 23, 24]. However, in extreme environments that urgently require system‐level simplification, such as aerospace, underwater exploration, and disaster rescue. These task‐specific solutions reveal fundamental limitations. Such scenarios demand systems capable of both withstanding the impact of dynamic, uncooperative targets and performing delicate manipulation on fragile static objects. Yet, they cannot afford the payload and spatial overhead required for bulky tool libraries, automatic tool changers, or multi‐arm setups. Furthermore, multi‐system collaborative planning is exceptionally challenging to implement, and the time latency and risk of mechanical failure associated with frequent interface switching in unpredictable environments are simply unacceptable. Currently, researchers have paid little attention to the fundamental limitations that prevent these single‐mode solutions from extending to opposite tasks. Specifically, the precise manipulation and low modulus materials of active soft hands lack the response speed and structural strength to handle fast dynamic targets. Meanwhile, the rapid clamping way of passive grippers cannot achieve universal shape adaptation or gentle capture, it also requires strict alignment of the target's incident direction with the gripper's trigger point. Thus, a fundamental contradiction exists between the two modes: softness versus rigidity in materials, rapidity versus slowness in response, and generalization versus precision in operation.

To overcome the critical risks of prohibitive spatial payloads and interface failures inherent to unstructured and tightly constrained environments, a handful of pioneering studies have recently explored cross‐modal grippers designed for the unified manipulation of both dynamic and static targets [12, 23, 24, 25, 26, 27]. Most of these efforts directly couple rigid multi‐link mechanisms with pneumatic joints. By adjusting the air pressure of these joints, they can program the stability characteristics of the gripper. This enables the gripper to handle both static and dynamic objects through active and passive modes, respectively. However, the rigid multi‐link sacrifices the critical advantages of compliance adaptation and continuous contact. Moreover, passive clamping solutions still remain at risk of impact damage and instantaneous rebound [28, 29, 30], as well as limitations in shape compatibility and stringent trigger conditions for incident directions [20, 22, 23, 24, 25, 27]. Ultimately, these cross‐modal grippers amount to a stacking of modes, inevitably leading to even deeper bidirectional performance degradation. Consequently, achieving an integrated coexistence of grasping modes and bidirectional performance optimization within a single soft gripper system continues to be a critical challenge.

**FIGURE 1:**
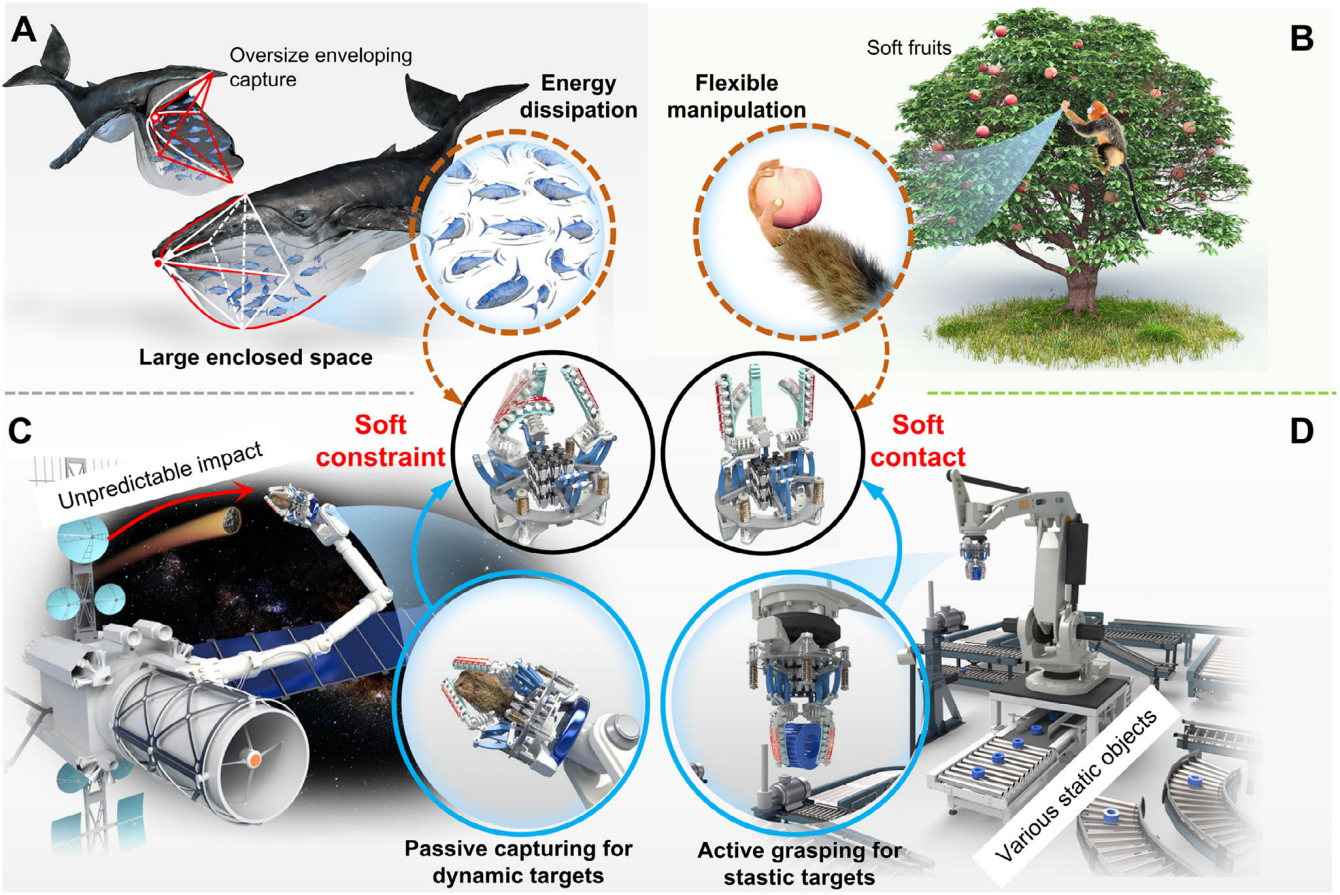
Design and strategy of the soft gripper inspired by predation. (A) Whales open their massive jaws to engulf seawater and schools of fish, achieving spatial confinement and energy consumption of prey after closing their mouths. (B) During the peach‐picking process, monkeys demonstrate excellent compliant grasping capabilities. When climbing, their hands apply substantial force to support body weight and grip hard branches, yet transition to compliant control during peach picking to prevent damage to the fruit. (C) In the cage configuration, the gripper expands the capture range. Utilizing the fingers' distinct properties in two directions, dynamic targets are made easy to enter but difficult to escape, achieving spatial confinement for passive capture. Simultaneously, the central metamaterial palm impacts with objects, providing effective energy absorption. This wide‐angle capture and energy‐handling capability is suitable for capturing uncooperative targets of unknown characteristics in space environments. (D) In a parallel configuration, the gripper mounted on a large industrial robotic arm could gently manipulate static targets of various shapes on assembly lines, offering high safety and compatibility.

Interestingly, the long process of natural evolution inspires us to treat grasping as a generalized form of predatory behavior. Creatures have evolved different predation methods according to their ecological niches [31, 32]. One way is contact predation, for example, humans, birds, and other animals directly exert force on prey with their limbs, achieving complex manipulation with precise control (Figure 1A). In contrast, creatures like gulper eels and whales utilize a soft constraining predation strategy that combines spatial confinement and energy dissipation (Figure 1B). They close their jaws to perform an oversize enveloping capture, encircling various prey within a firm enclosed space. The trapped prey exhausts its energy through the struggles and collisions inside.

Rather than replicating the exact structures or behaviors of organisms, this work strategically fuses the essential wisdom of two soft predation mechanisms into a single soft gripper [3, 4, 31, 32]. First, the designed gripper adopts a parallel configuration to realize the soft contact mechanism for active grasping static targets [2, 5, 7, 8, 13, 14, 15, 16, 17, 18, 19], and a cage configuration to realize the soft constraint mechanism for passive capturing dynamic targets [28, 29, 33, 34]. Based on the simple strategy [35, 36, 37, 38, 39] that employing dual‐mode morphological configuration to decouple cross‐modal grasping, we resolve the conflicts between the two grasping modes and employ different soft mechanisms in distinct configurations. After achieving this decoupling, we specifically design a soft finger and a palm, which can satisfy the requirements of two mechanisms and handle the various characteristics of the target objects (Figure 1C,D). Serving as the foremost execution component, the soft finger integrates the exoskeleton–tendon composite structure. It not only optimizes active output for compliant grasping, but also possesses stiffness anisotropy [33, 34] and monostable characteristics [40, 41, 42, 43], both of which contribute to its unique passive functionality. The metamaterial palm hierarchically combines two classical structures [44, 45, 46, 47], a rigid snap‐fit [48, 49, 50] and a soft buckling beam [51, 52, 53, 54]. The palm achieves subsequent processing for energy through alternating responses with multistep stiffness damping and reversible multistable deformations [44, 45, 46, 47, 49, 51, 52, 53, 54]. Building upon these components, we construct a core morphological mechanism that enables ingenious reconfiguration of the two shape modes within a unified architecture. This approach achieves top‐level coordination of finger and palm, going beyond a simple linear superposition of parts. In the parallel configuration, the soft finger's superior active output enables gentle grasping to static targets. In the cage configuration, leveraging the finger's passive characteristics on stiffness and position, the gripper rapidly imposes spatial confinement on incoming dynamic targets. Concurrently, the palm instantaneously executes highly efficient energy handling and rebound suppression at the moment of impact within the cage. The finger and palm together implement a soft‐constraint capture, providing combined handling both in spatial and energy domains.

Moreover, we establish a morphological model to simulate and optimize the entire process of configuration switching [38], thereby enhancing the independent effectiveness of each soft mechanism. Through this top‐level synergy, we distinctly utilize the properties of each component for different target types and achieve bidirectional gains in grasping performance.

Extensive experiments show that the proposed soft gripper achieves a balance between mode compatibility and functional decoupling in one integrated system [55, 56, 57, 58, 59, 60, 61, 62]. It retains the inherent advantage of compliance through soft contact during active grasping of static objects. In passive capture of dynamic objects, the coordinated finger–palm constraint mechanism extends the passive capture to uncooperative targets under highly dynamic conditions and uncertainties in relative locations and velocities. In addition, the gripper demonstrates storage and transfer of multiple heterogeneous targets simultaneously [17]. The proposed gripper demonstrates compelling advantages in extreme environments, such as space exploration, deep‐sea operations, and disaster rescue [30, 33, 34]. These scenarios strictly require robotic systems to rapidly and reliably intercept uncooperative targets subjected to high‐speed falls or turbulent impacts, while simultaneously performing delicate manipulation on static, fragile objects. In contrast to conventional paradigms that rely on an array of task‐specific grippers, our design achieves system‐level simplification through functional integration. Consequently, it drastically alleviates the control complexity and systemic overhead inherently associated with multi‐task coordination. This work broadens the performance and application boundaries of soft robotic grippers and serves as a valuable paradigm for the exploration of cross‐modal soft grasping.

## 2 Results

### 2.1 Composite Soft Finger with Integrating Exoskeleton‐Tendon System

**FIGURE 2:**
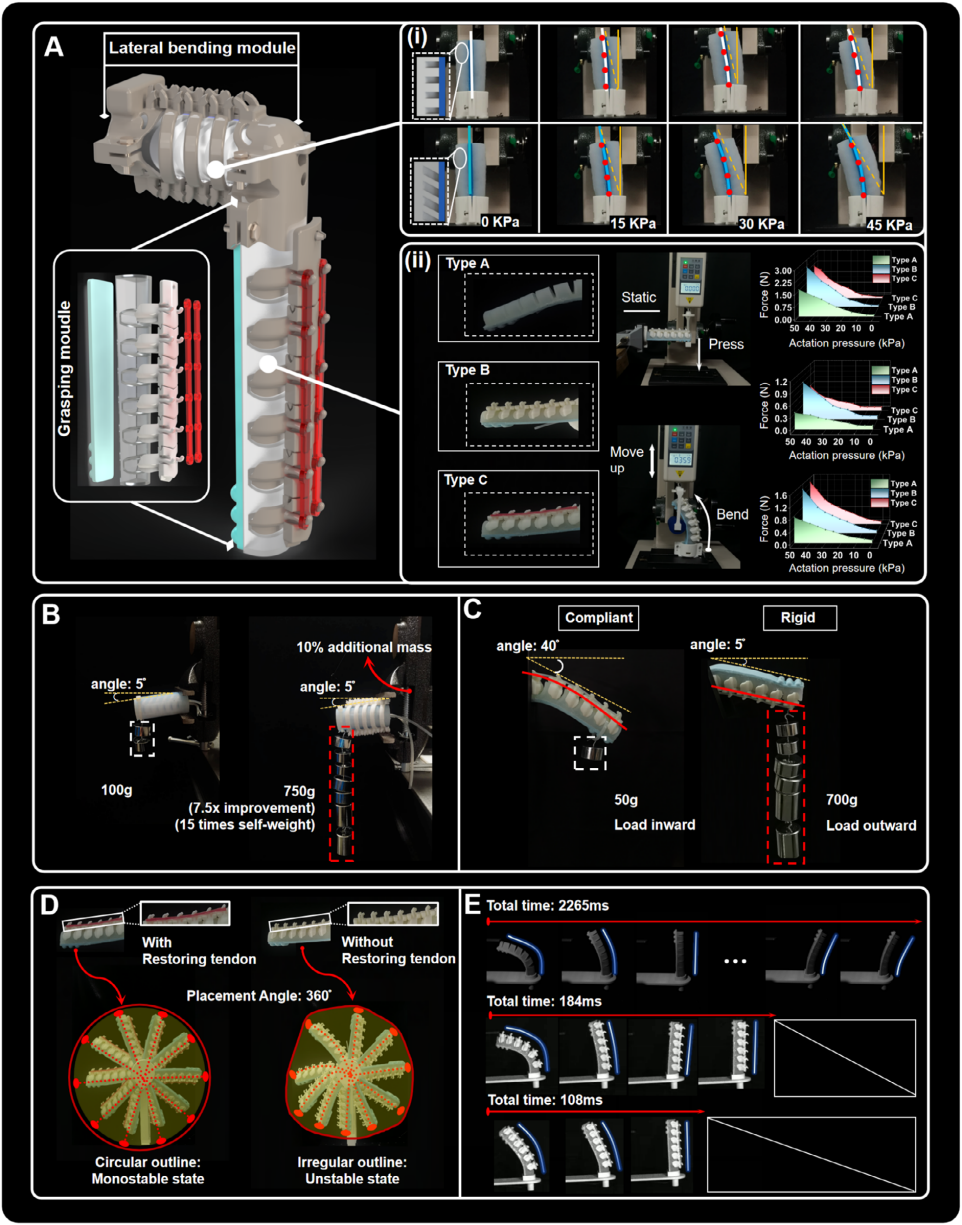
Design and performance characterization of the soft finger. (A) The soft finger consists of a lateral bending module and a grasping module. (i) The lateral bending module's soft actuator uses axial internal chambers (top) and diagonal chambers (bottom), showing a comparison of sideways bending under the same pressure. (ii) Performance output comparison of three grasping modules: from left to right, first column shows a normal tapered module (Type A), a tapered module with rigid exoskeleton blocks (Type B), and a module with exoskeleton blocks and an elastic tendon (Type C). The second column shows the measurement setup and method. The third column presents the experimental results for each finger type in the first column (top to bottom: grasping force, contact force, and fingertip force). (B) Comparison of longitudinal load ability of the lateral bending module, before and after adding the exoskeletal sheath. (C) Comparison of stiffness between the ventral and dorsal sides of the grasping module. (D) Monostable pose enabled by the elastic tendon. (E) Comparison of return times for the three types of grasping module (top to bottom: Type A, Type B, and Type C); the white diagonal box highlights the time saved relative to Type A.

As the direct execution component for grasping, the soft body is directly responsible for soft contact grasping in active mode and soft constraint capture in passive mode. As mentioned above, to satisfy different requirements under different configurations, we integrate an exoskeleton and tendon system into fingers. Each finger is composed of a lateral bending module and a grasping module, which are orthogonally arranged in series (Figure 2A), providing lateral and inward degrees of freedom to achieve human‐like grasping and pinching. The lateral bending module is composed of a soft actuator featuring oblique pneumatic chambers, which is encased in a rigid exoskeletal constraint sheath. The grasping module integrates a multi‐layer structure: a constraint layer, a tapered deformation layer with concave pneumatic chamber, complemented by rigid exoskeletal blocks and an elastic tendon (detailed in Figure S1 and Text S2).

For output optimization, compared to the conventional axial chamber design, the oblique chambers (30°) enhance lateral actuation capability (detailed in Figure S3 and Text S1). Under 45 kPa driving pressure, the lateral bending angle reaches 45°, nearly doubling 200% that of an axial‐chamber actuator (Figure 2Ai). The grasping module improves its force output by alternating concave soft pneumatic chambers and convex rigid blocks. To clear the influence of this soft‐rigid hybrid structure, we create an analysis model (detailed in Text S1).

(1) $$\theta =\frac{l}{h^{\prime }+r}=\frac{\eta_{rigid}l_{0}+\left(1+\varepsilon_{cham}\right)\left(1-\eta_{rigid}\right)l_{0}}{h^{\prime }+r}$$

(2) $$F_{\mathrm{tip}}=\sum_{i=1}^{n}\frac{\int \sigma_{i,y,e}dA_{\mathrm{xz}}+l_{i}\sin\theta_{i}\int \sigma_{i,x}dA_{\mathrm{yz}}+l_{i}\cos\theta_{i}\int \sigma_{i,y}dA_{\mathrm{xz}}}{l_{i}\cos\theta_{i}}$$

where *l_0_* and *l* are the initial length and longitudinally elongated length of the grasping module, *ε_cham_* is the longitudinal strain of the chamber, which is increasing with *η_rigid_*, *θ* is the bending angle of the module, *h’* is the height where the maximum longitudinal strain occurs, *r* is the curvature radius of the bent module, and *η_rigid_* is the ratio of rigid structures. *σ_i_* and *dA* denote the stress and contact area between the soft and rigid components, respectively. *σ_ix_* and *σ_iy_* are the components of *σ_i_* in *x* and *y* directions, and *σ_i,y,e_* is the component of *σ_i_* in vertical direction, representing the additional stress applied by the curved interface between soft and rigid materials. The bending angle of the grasping module is positively correlated with the rigid‐structure ratio *η_rigid_*. Increasing *η_rigid_* appropriate can increase the bending angle. The rigid block covering the top of the soft chamber introduces an additional bending moment, further boosting the fingertip force. Moreover, the tapered shape with a gradually shrinking cross‐module optimizes the enveloping capability of the grasping module (detailed in Figure S4 and Text S1). The module also exhibits a bending curvature that decreases toward the tip. This design yields a larger contact area on objects through the large surface curvature (2), while a smaller fingertip can pinch small targets. Figure 2Aii shows the characterization of the grasping module's output. Compared to the standard design (Type A), the improved soft–rigid coupled design (Type B) boosts fingertip force and enveloping force up to 200%, and contact force up to 300%. Adding an elastic tendon (Type C) slightly reduces the force, which can be compensated by modestly increasing the driven pressure.

The integrated exoskeleton endows the soft finger with an anisotropic stiffness profile, without compromising the degrees of freedom provided by individual segments, thereby fully preserving the dexterity of active grasping. (detailed in Figures S1 and S2). In the lateral direction, the sheath exhibits a phase‐angle‐dependent stiffness modulation, effectively suppressing the seal failure due to over‐inflation and preventing buckling instability under high load. When the bend angle is below a threshold *θ*, the sheath exhibits no resistance to actuator movement, preserving finger compliance. When the angle is beyond *θ*, interference from the transverse protrusions of sheath block any further increase in bending angle, even if the actuator is over‐pressurized. The total bending angle satisfies:

(3) $$\theta =\sum_{i=1}^{n}\theta_{i}$$

With only a small added mass (100 g), the sheath's spinous processes constraints yield a 750% increase in longitudinal load capacity. This enhanced capacity is critical for grasping stability (Figure 2B). In the grasping module, rigid blocks do not constrain inward bending but form a mortise‐and‐tenon interlock to resist outward bending. As shown by experiments in Figure 2C, the grasping module's stiffness between inward bending (holding 50 g at 40° deflection) and outward bending (700 g at 5° deflection) differs up to 11 200%. Thus, the finger meets the disparate stiffness requirements needed for different grasping modes.

Like a human finger, the elastic tendon gives the finger passive monostability on position. Without any additional energy or complex control, the finger can maintain a straight posture in any direction (360°). It can also rapidly return outward to its initial shape after being bent inward by an impact (detailed in Figure S5 and Text S2). Leveraging this property, the caging gripper can rapidly establish a stable spatial enclosure once a dynamic target enters. Under dynamic impacts, the bending angle and the recovery speed of the grasping module are critical for successful capture. Hence, we develop a theoretical model for the large deformation bending process of the grasping module (detailed in Figure S5–S7 and Text S2).

(4) $$\theta_{i}=\frac{2r_{b}x_{bi}}{{x_{bi}}^{2}+{y_{bi}}^{2}}$$

(5) $$\theta_{i+1}=\theta_{i}+{\dot{\theta }}_{i}\Delta t+\frac{1}{2}{\ddot{\theta }}_{i}\Delta t^{2}$$

(6) $${\dot{\theta }}_{i+1}={\dot{\theta }}_{i}+{\dot{\theta }}_{i}$$

where *r_b_* is the length of the grasping module, *θ_i_* and *θ_i+1_* are the fingertip deflection angles at time steps *i* and *i+1*, ${\dot{\theta }}_{i}$ is the tip's angular velocity at time *i*, ${\ddot{\theta }}_{i}$ is the tip's angular acceleration at time *i*, and *x_bi_* and *y_bi_* are the position of the center of the grasping module. Through the grasping experiments, we confirm that selecting a polyester elastomer with Shore hardness 00‐30 for the elastic tendon provides a sufficient bending angle for target entry. In comparative tests with three finger prototypes, once external constraints are removed, the elastic tendon releases its stored energy within 108 ms, allowing the soft finger to rapidly rebound and complete the capture (Figure 2E, Movie S1). The finger in type C achieves a 95% and 41% reduction in recovery time relative to Finger A and B, respectively.

The introduction of rigid blocks reinforces the finger's directional load‐bearing capabilities without restricting its intrinsic degrees of freedom. Through the above design optimizations, the soft finger exhibits excellent compliant output performance in a parallel configuration. Moreover, its anisotropic stiffness and monostable shape characteristics lay an important foundation for achieving generalized spatial confinement in cage configuration.

### 2.2 Multi‐Level Palm Design for Rebound Suppression and Energy Dissipation

Although the soft finger enables a gripper to impose spatial constraints on dynamic targets, it cannot similarly realize the crucial energy management capability inherent to wide‐range enveloping predation independently. It is noteworthy that this capability remains a universally lacking but critically needed feature in current soft gripper designs, especially for dynamic capture. Collisional energy easily destroys both the grasped object and the rigid palm. If it is a soft palm, the viscoelasticity of the soft material may act as a booster for the object to escape [29]. Here, we design a metamaterial palm with a unique multi‐level alternating rigid‐soft response, filling this functional gap that cannot be covered by fingers alone.

**FIGURE 3:**
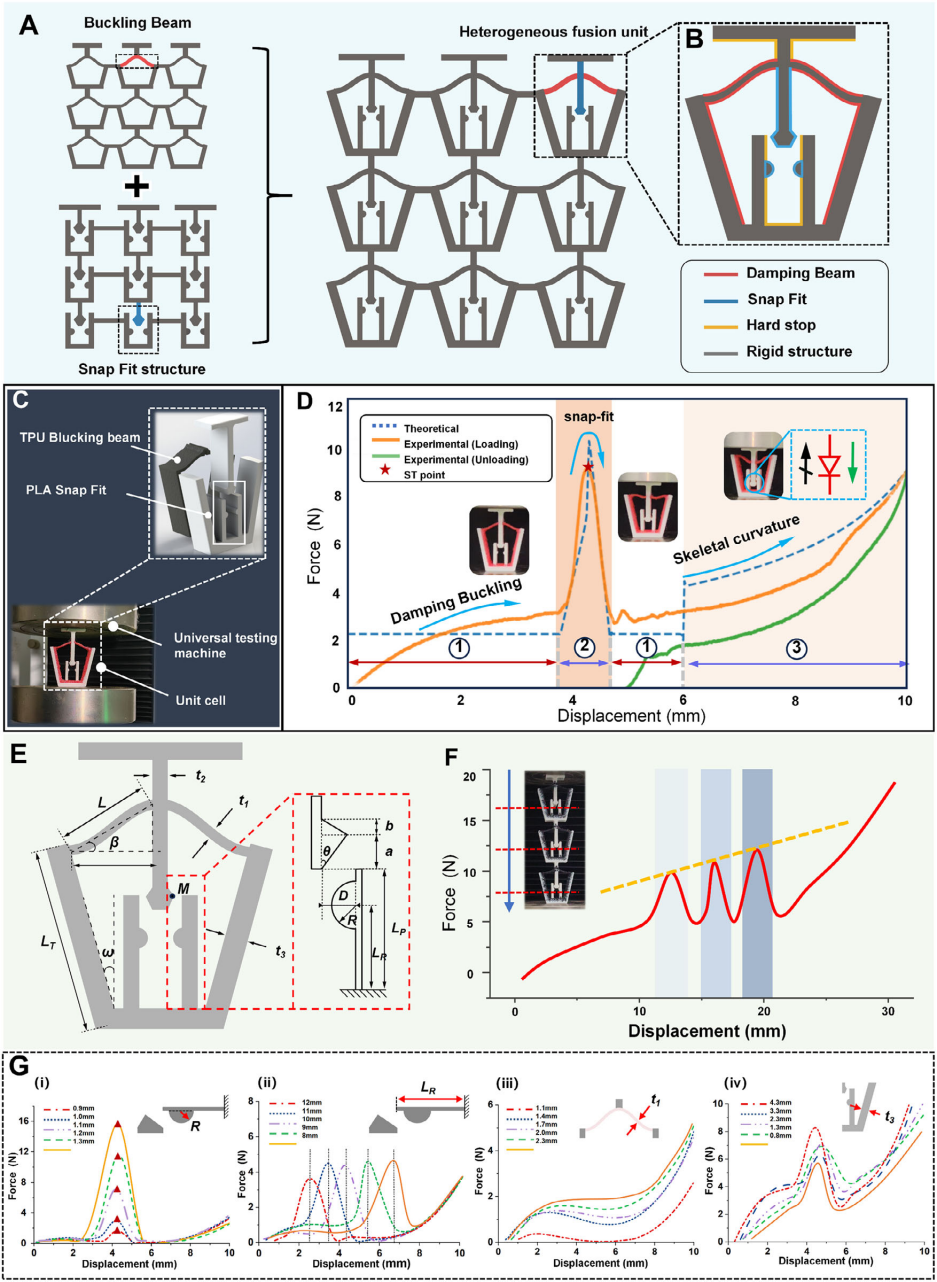
Design of biphasic metamaterial cell unit. (A) The integration mechanism of buckling beams and snap‐fit structures. (B) Detailed structure of the metamaterial unit cell. (C) The upper panel is the quasi‐static compression test, lower panel is the dual‐material composition of the unit cell. (D) Theoretical response of a typical unit cell under loading, performed in three distinct segments, depicted by blue dashed lines; actual response curves show loading (orange solid line) and unloading (green solid line) processes. (E) Key parameter model of the metamaterial unit cell, with the snap‐fit structure enlarged within the red dashed box. (F) Hierarchical programmable response curve of the metamaterial unit cell. (G) Programming the unit cell response through key parameter design. (i) shows the programmed response curve for the snap‐fit radius *R*; (ii) shows the programmed response curve for the snap‐fit characteristic length *L_R_*; (iii) shows the programmed response curve for the beam thickness *t_1_*;(iv) shows the programmed response curve for the frame thickness *t_3_*.

Specifically, using multi‐material fused deposition modelling, we integrate two classical structures, a flexible buckling beam and a rigid snap‐fit, into a metamaterial unit cell (Figure 3A). The buckling beam (TPU) serves as a soft phase that provides large‐displacement damping and cushioning, while the snap‐fit (PLA) provides large energy absorption at small displacements. The support frame (PLA) serves constrained blocking with a negative Poisson's ratio characteristic (Figure 3B). This biphasic cell combines the key benefits of two phases, overcoming limitations in single‐material palms.

We first conduct quasi‐static compressive tests (Figure 3C). The response curve exhibits four distinct stages (Figure 3D), which can be effectively described by three theoretical models (blue dashed lines, numbered 1, 2, and 3 in Figure 3D) (detailed in Figures S9–S11 and Texts S3–S5). During Stage 1, the soft beam undergoes compressive buckling, dissipating energy via viscous damping. Upon loading into Stage 2, the rigid snap‐fit exhibits a singular peak at the snap transition point (ST point). This arises because the rigid‐phase material absorbs significantly more energy than the soft‐phase material for the equivalent elastic deformation. When the load exceeds 9.3 N, the unit cell completes its entire deformation process. With the integration of the snap‐fit, energy absorption increases by about 212% over a short stroke, and the peak force is amplified nearly 300%. Upon deformation completion, the curve maintains the characteristics of the soft‐phase beam in Stage 1. The unit exhibits unstable deformation and maintains a concave state [44]. Although it is bistable, it can easily lose stability when subjected to even minor disturbances. At this point, the locking action of the snap‐fit prevents the unit from recovering its original state, thereby preventing the object regaining energy from the palm's elastic potential energy. Continuously applying load to reach Stage 3, the result reveals a response curve dominated by the compression of the flexible beam. The beam pulls the rigid exoskeleton into buckling until constrained by the hard stop limit. Throughout this process, the stiffness of the unit increases progressively, and the slope of the curve rises continuously, ultimately reaching the second peak. During unloading, the unit retains its shape (see Movie S2). This retention of unrecovered deformation is what gives the unit excellent energy absorption properties. The snap‐fit acts like a mechanical diode, permitting downward displacement while resisting recovery upward, thus ingeniously resolving the challenging issue of shape restoration.

We establish an analytical model of the unit cell for extracting key parameters, they are soft‐phase beam thickness *t_1_*, buckling radius *R*, buckling characteristic length *L_R_*, and frame thickness *t_3_* (Figure 3E). Hierarchical array configuration of the unit cell (Figure 3F) enhances the energy absorption range. Figure 3G presents experimental results where parameters are varied to tailor the response of a single cell. The value of the energy barrier and the emergence position of snap‐fit can be independently controlled: increasing *R* can enhance the value of absorbed energy and energy barrier (G(i)); while maintaining the proportional factor *R* / L_R_^3^ constant, the position of the peak point can be controlled by adjusting *L_R_* (G(ii)). The increase in thickness *t_1_* results in an enhanced reaction force of the soft beam and realizes a transition of the stiffness response from negative to positive (G(iii)). Correspondingly, increasing the skeleton thickness *t_3_* enhances stiffness after the peak, while driving the response from positive to negative stiffness (G(iv)). When the thickness ratio *t_3_* / *t_1_* falls within the range of 1.3 to 4.0 [51, 52], the unit cell exhibits a quasi‐zero stiffness condition (detailed in Texts S3–S5 and Movie S2). Considering the interference between beam and snap‐fit, while maintaining *t_3_* constant, we selected *t_1_* values of 1.7, 2.0, and 2.3 mm for the subsequent design. Experiments on multi‐layer unit cells (Figure 3F) confirm that altering predefined parameters can also induce controlled sequential responses. Here, the sequence exhibits a sequential response from the top to down. The three peak potential barriers perform an increasing relationship.

**FIGURE 4:**
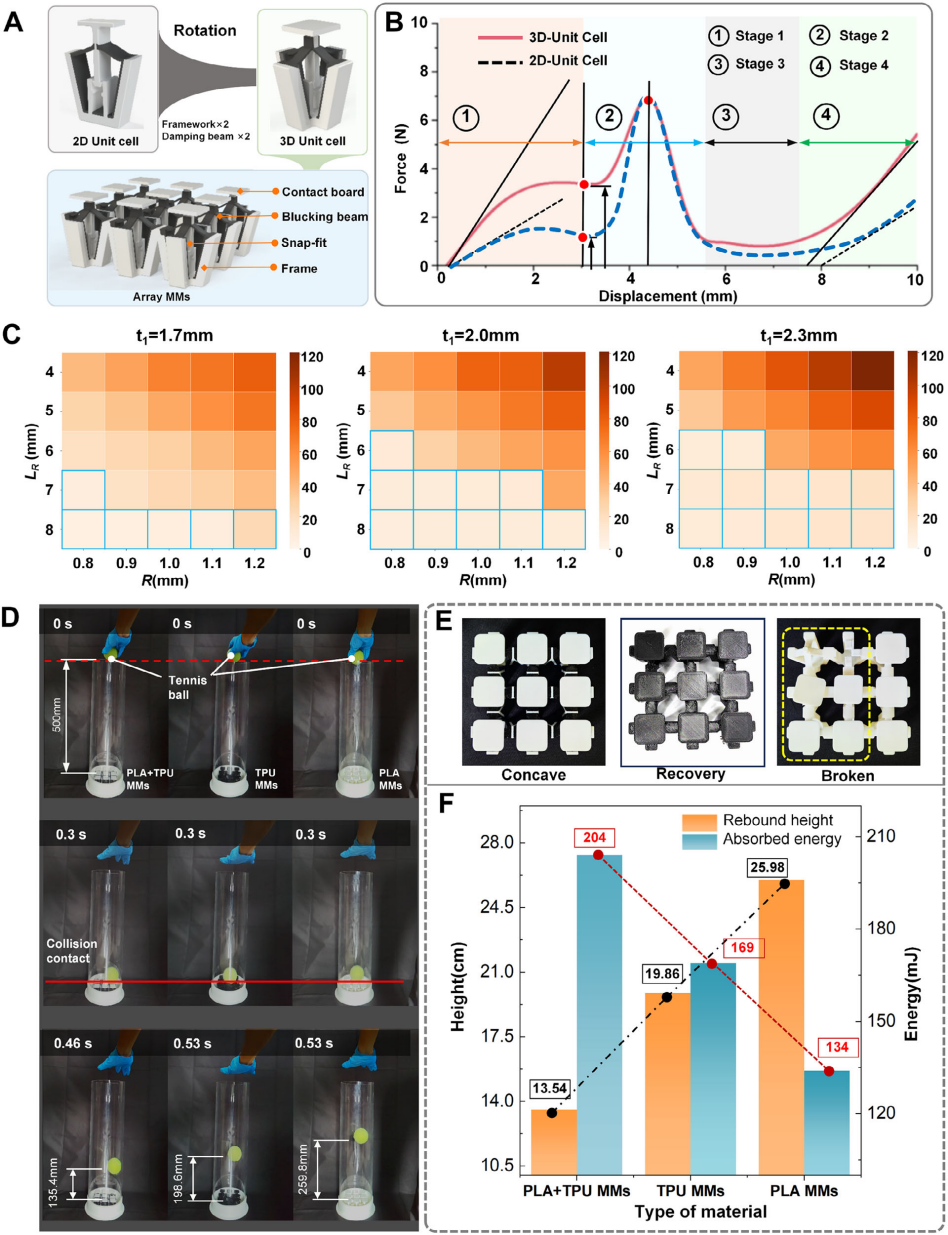
Hierarchical design and characterization of the metamaterial palm. (A) 3D cell constructed by a 90° rotation of the 2D cell. An array of such cells forms a single‐layer 3D metamaterial palm. (B) Comparison of response curves between the 2D and 3D cells, the 3D cell shows roughly doubled response in the first and fourth stage. (C) Heat maps of energy absorption for 3D cells with different beam thickness. (D) Energy absorption experiment for single‐layer palms in different kinds of material. The ball falls freely from the air and falls onto the metamaterial (MMs). From left to right: a biphasic palm of PLA‐TPU (structure intact and shape retained after impact), a TPU palm (structure intact but shape recovered), and a PLA palm (structure damaged). (E) Outcome for the three palms after impact are intact and stable shape (PLA‐TPU palm), intact but reverted shape (TPU palm), structural damage (PLA palm). (F) Comparison of rebound heights after impact and energy absorption for the three types of palms.

Building upon the two‐dimensional unit cell, beams and frames are rotated 90° to construct a three‐dimensional unit cell with a spatially orthogonal distribution. Subsequently, the three‐dimensional unit cells are arranged in a periodic array, yielding a single‐layer palm (Figure 4A). In this way, we broaden the energy absorption range of the single‐layer palm. The top contact plate can rotate in three‐dimensional deflection, providing an optimal impact surface. Theoretically, the unit cell inherits the response of snap‐fit and redoubles the reaction forces of both the soft beams and support frame, achieving significant damping and cushioning. Quasi‐static compressive experiments conducted in Figure 4B validate this theory. Experimental data for varying characteristic parameters of the three‐dimensional unit cell are presented, with heat maps of energy absorption for three t1 values shown in Figure 4C. Energy absorption increases with rising R and decreasing LR, while also showing a positive correlation with increasing thickness t1. Within the deformation retention failure zone (denoted by the blue box), energy absorption markedly diminishes. This phenomenon may be attributed to the meshing force of the snap‐fit being insufficient to counteract the reaction force of the buckling beam, which results in a loss of morphological stability upon disturbance. Therefore, given that the snap‐fit is securely locked, the optimal strategy for enhancing energy absorption is to increase *R* and decrease *L_R_*.

To further evaluate the performance, a contrast experiment is conducted on the single‐layer metamaterial array (Figure 4D). A tennis ball is released freely from 500 mm, and its rebound height is observed. Experiments demonstrate that the biphasic metamaterial exhibiting outstanding energy absorption properties, with structural integrity and stable morphology after impact (Figure 4E). As shown in Figure 4F, compared to conventional rigid‐phase or soft‐phase palms, its energy absorption increases to 153% (PLA) and 121% (TPU) respectively, cutting the rebound distance by nearly 50% (see Movie S2). Based on these results, we implement hierarchical programming for the palm. The top layer (contact layer) needs low energy barriers of snap‐fit and negative stiffness properties, enabling more sensitive responses and primary absorption of energy. The middle layer serves as a buffer structure with moderate energy barriers and quasi‐zero stiffness characteristics. The bottom layer incorporates buckling beams in positive stiffness and the highest energy barriers to withstand high‐energy impacts, providing final resist while offering stable support to the upper two layers.

In response to the challenge of impact energy in cage configurations, we have correspondingly developed a metamaterial palm module for back‐end processing. Through the finger‐palm coordination, it provides critical assurance for the caging gripper's effective performance in both spatial and energy dimensions as well as an effective non‐destructive countermeasure.

### 2.3 Implementation and Optimization of Dual‐Mode Configuration and Switching

**FIGURE 5:**
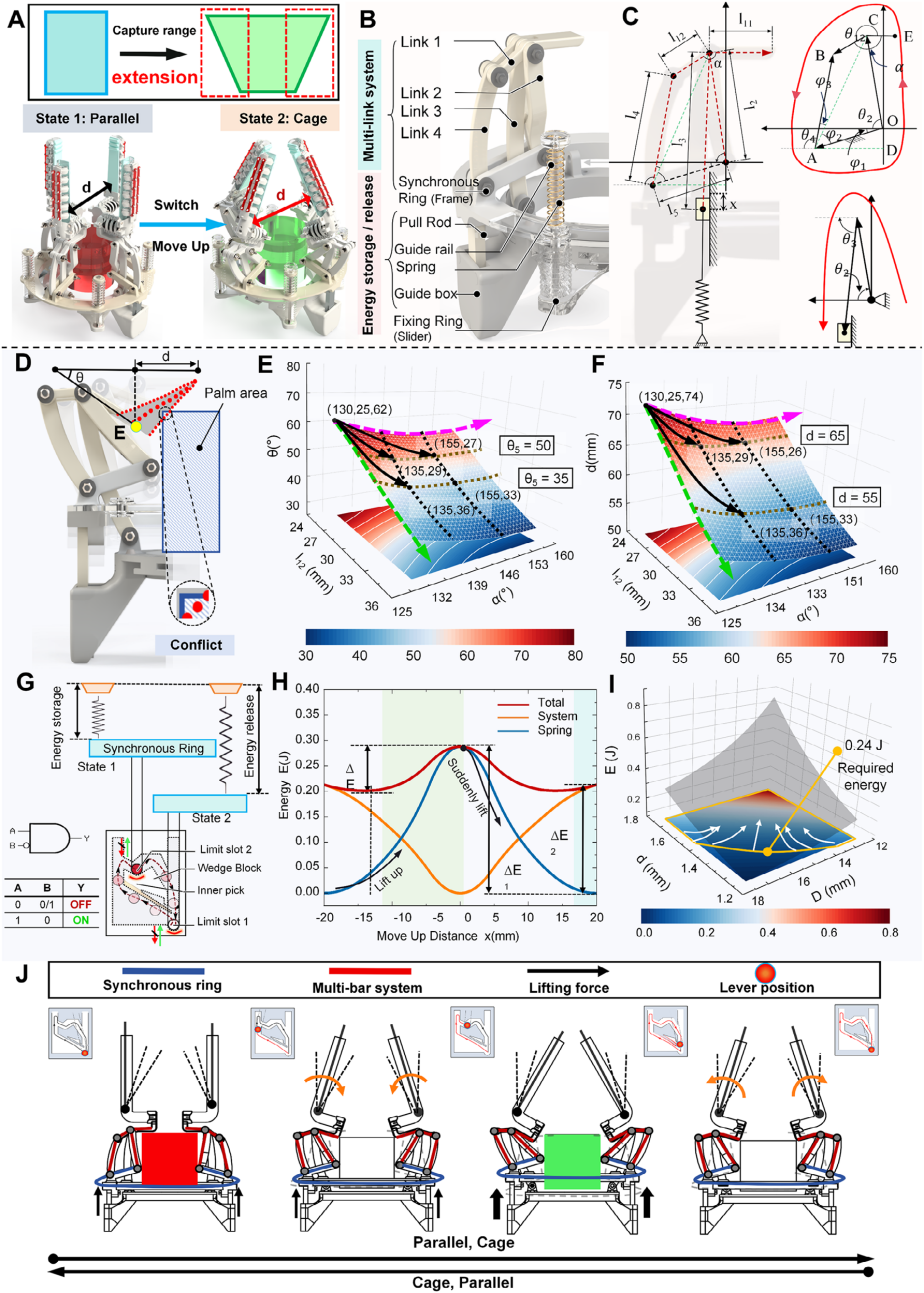
Dual‐morphology switching mechanism based on a switching frame. (A) Lifting the synchronous ring transforms the gripper from the parallel configuration to the cage configuration. The red cylinder indicates the palm is hidden (inactive), while the green indicates the palm is revealed (active). The upper graph shows that the effective capture range of caging gripper is significantly wider than that of conventional rapid gripping. (B) The switching frame consists of a multi‐link system, an energy storage, and the release system. (C) Model strategy based on dual‐loop domain decoupling. The top‐right shows the isolated first loop, and the bottom‐right indicating the isolated second loop. (D) Rotation angle provided to the fingers by the switching frame and the change in distance of opposing fingers, along with the trajectory of the inner feature point. (E) Effect of abduction length and abduction angle on the rotation angle. (F) Effect of abduction length and abduction angle on the change in distance of the inner feature point. (G) The position of synchronous ring before and after energy release. The red ball inside the guide box indicates the path of pull rod. The guide box mechanically determines the trigger force direction, functioning as a logic gate. (H) Energy variation of the system over one switching cycle. (I) Relationship between wire diameter, mean diameter, and energy of the spring. (J) Changes in the gripper's morphology over one switching cycle. Red squares indicate the palm is inactive, while green indicates the palm is deployed and collaborating with the fingers for capture. The top‐left shows the pull rod's position inside the guide box.

Not like to existing cross‐modal grippers that stack functional components linearly, which suffer performance trade‐offs, we introduce morphological reconfiguration within an integrated architecture. This strategy fundamentally departs from the paradigm of using a single morphology to reconcile the conflicting demands of dual modalities. By exploiting closed and non‐enclosed structural configurations, we strategically map the parallel form and cage form to opposite grasping modes, and leverage the soft fingers and metamaterial palm to support the corresponding grasping mechanisms. In this way, we achieve genuine cross‐modal performance optimization. Notably, tailored to the specific demands of diverse application scenarios, this strategy can be readily extended to alternative geometric configurations with corresponding enclosure characteristics, exhibiting exceptional versatility and potential. As illustrated in Figure 5A, in the parallel configuration (I), the soft fingers are arranged parallel to each other, and the palm does not need to act. In the cage configuration (II), the soft fingers converge at a point, while the palm cooperate with the fingers to accomplish efficient capture. Grasping dynamic targets with a traditional approach often entails intense opposing forces. The cage configuration, by contrast, uses a novel soft constraint method instead of a rapid clamping, achieving generalized spatial confinement and energy modulation that conventional gripper cannot attain.

Therefore, the switching frame must enable the fingers to control whether the palm's contact surface is exposed, thus allowing on‐demand coordination between fingers and palm. Effectively utilizing the differing characteristics of the soft finger and the palm in each configuration is a key consideration for optimizing the design. Ideally, the finger spacing should be reduced in parallel mode to ensure a secure grasp, and expanded in cage mode to accommodate dynamic targets of various sizes and shapes. In addition, adequate finger rotation is needed to alter the force transmission path, enabling flexible reuse of the soft fingers’ properties. To enable a highly precise, complex, and coupled rotation and translation motion of the fingers, it necessitates a reconfiguration capability far more sophisticated than simple single‐pivot rotation.

Based on these insights, we design the switching frame shown in Figure 5B (detailed in Figure S12 and Text S6). Four fingers are connected via a circumferentially distributed multi‐link system (links 1 to 4) to both the synchronous ring and fixed ring. By lightly toggling the synchronous ring upward, only a small lift displacement is needed to achieve a large morphological transition. The energy system, consisted of a self‐locking mechanism and a spring‐slider rail, can store and release elastic potential energy within 0.2 s. It fulfills the energy allocation required for both forms without requiring additional complex drives.

We construct a detailed kinematic model to simulate the entire shape‐switching process of the gripper. To tackle the complex multiple nonlinear couplings in the system, we propose a model strategy based on dual‐loop decoupling (detailed in Figure S13 and Text S6), which provides an efficient analytical framework for the complex mechanism's motion. By treating the synchronous ring as fixed, the original model is transformed into a coupled system with crank‐slider and double‐rocker, enabling the establishment of a dual closed‐loop vector model (Figure 5C).

(7) $$\begin{array}{c} l_{2}e^{i\theta_{2}}+l_{12}e^{i\theta_{12}}=l_{5}e^{i\theta_{5}}+l_{4}e^{i\theta_{4}} \end{array}$$

(8) $$\begin{array}{c} l_{2}e^{i\theta_{2}}+l_{3}e^{i\theta_{3}}=l_{6}e^{i\theta_{6}} \end{array}$$

In the subsystem, we derive the motion equations for key points by strategically introducing a time‐varying auxiliary line AC and a fixed auxiliary line AD. Along with geometric constraints, the initial model is simplified accordingly:

(9) $$\vec{l_{2}}+\vec{l_{12}}=\vec{l_{5}}+\vec{l_{4}}$$

(10) $$\left[\begin{array}{c} l_{2}\cos\theta_{2}\\ l_{2}\sin\theta_{2} \end{array}\right]+\left[\begin{array}{c} l_{12}\cos\theta_{12}\\ l_{12}\sin\theta_{12} \end{array}\right]=\left[\begin{array}{c} l_{5}\cos\theta_{5}\\ l_{5}\sin\theta_{5} \end{array}\right]+\left[\begin{array}{c} l_{4}\cos\theta_{4}\\ l_{4}\sin\theta_{4} \end{array}\right]$$

(11) $$\vec{l_{2}}+\vec{l_{3}}=\vec{l_{6}}$$

(12) $$\left[\begin{array}{c} l_{2}\cos\theta_{2}\\ l_{2}\sin\theta_{2} \end{array}\right]+\left[\begin{array}{c} l_{3}\cos\theta_{3}\\ l_{3}\sin\theta_{3} \end{array}\right]=\left[\begin{array}{c} x_{1}\\ -H-\Delta x \end{array}\right]$$

where $\vec{l_{2}}$ is a bridging element since the two subsystems are coupled through the rotation of link L2. We establish a non‐affine mapping between the dual‐loop inputs (*x*, *θ_2_*), ultimately yielding a dual‐loop decoupled model of the shape‐switching system.

(13) $$l_{2}\cos\theta_{2}+l_{3}\cos\theta_{3}=x_{1}$$

(14) $$l_{2}\sin\theta_{2}+l_{3}\sin\theta_{3}=-H-\Delta x$$

(15) $$\vec{OE}={\vec{l}}_{5}+\vec{l_{4}}-\vec{l_{12}}+\vec{l_{11}}=\left[\begin{array}{c} x_{1}+x_{2}\\ -H \end{array}\right]+\left[\begin{array}{c} l_{4}\cos\theta_{4}\\ l_{4}\sin\theta_{4} \end{array}\right]-\left[\begin{array}{c} l_{12}\cos\theta_{12}\\ l_{12}\sin\theta_{12} \end{array}\right]+\left[\begin{array}{c} l_{11}\cos\theta_{11}\\ l_{11}\sin\theta_{11} \end{array}\right]$$

The finger rotation angle (*θ_5_*) and distance variation (d) constitute the core parameters of configuration (Figure 5D). After assigning values to necessary variables according to design requirements, the system retains only three undetermined variables: *l_4_*, *α*, and *l_12_*. It is keenly observed that *l_4_* is a redundant variable determined by *α* and *l_12_*. This discovery simplifies the system to a binary optimization model concerning on *α* and *l_12_*, with the results are presented in Figure 5E,F. Figure 5E shows the response surface of rotation angle *θ_5_* with respect to abduction length *l_12_* and abduction angle *α*. The pink dashed line indicates that increasing *α* reduces *θ_5_* when *l_12_* is held constant. The green dashed line shows that increasing *l_12_* similarly decreases *θ_5_* when *α* is fixed. By comparing the decay rates of the two dashed lines and the contour lines of the surface, we find that the sensitivity of *θ_5_* to *l_12_* is significantly higher than that to *α*. Based on this, starting from the assumed initial value point, *l_12_* is optimized within the required deflection angle range (35°–50°). Subsequently, tuning is performed within the reasonable range of *α* (135°–155°). The feasible domain for the rotation angle (*l_12_*, *α*) is enclosed by the boundary curve (black dashed line). Figure 5F indicates that the distance (*d*) similarly exhibits a negative correlation with both variables, while greater sensitivity to abduction length. Similarly, the feasible domain is determined within the target distance range (55–65 mm). The intersection of the feasible domains in figures defines the theoretical scope for feasible design.

The end‐point E of link 1 must fully deploy the palm while ensuring its trajectory never intersects the palm. Owing to the circumferential symmetry of the system, we can analyze E's reachable trajectory in a representative 2D cross‐section (Figure 5D). However, although the parametric pairs differ only at millimeter scale, they can still result in various morphological anomalies. Based on our theory, we developed a visualization of the entire switching process. The excellent agreement with scaled‐down experimental results the analytical model is robust and precise, which can provide a reliable foundation for design. (see Movie S3 and detailed in Figures S14–S19 and Text S6). Ultimately, we chose the rotation length (30 mm) and rotation angle (150°) corresponding to the center of the feasible region as the design values for the mechanism.

We further model the energy system (detailed in Text S7). Figure 5H shows the energy variation over one switching cycle. The total potential energy of the gripper (*U_total_*, red curve) is the sum of the switching potential energy of the multi‐link system (*U_system_*, orange curve) and the potential energy of the spring (*U_spring_*, blue curve):

(16) $$\begin{array}{ccc} & & U_{total}=U_{systeml}+U_{spring}=\\ & & \sum \left(m_{i}gh_{i}\right)+4\sum \left(m_{0}gh_{0}\right)+m^{\prime }gh^{\prime }+\frac{1}{2}K\Delta x^{2} \end{array}$$

where *m_i_* is the mass of each link, *h_i_* is the vertical height from the zero‐potential‐energy plane to the center of mass, g is the gravitational acceleration, *m_0_* is the mass of each finger, *h_0_* is the vertical height from each finger's center of mass to the zero‐potential‐energy plane, *m'* is the mass of the synchronization ring, *h'* is its vertical height to the zero‐potential‐energy plane, *K* is the compression spring stiffness, and *∆x* is the slider displacement. It is noteworthy that the guide box functions as a mechanical logic gate, intelligently discerning trigger signals, and operates without incorporating additional actuators (Figure 5G). Switching is activated solely by upward lifting of the synchronous ring; downward pressure aligned with the load direction fails to trigger it.

The variation in the sub‐energy system is crucial for switching and configuring the morphology (Figure 5H). *U_spring_* manifests as a shape‐symmetric convex parabola, with its peak at x = 0 mm (steady state I, cage state) and its trough at x = ±14 mm (steady state II, parallel state). *U_system_* and *U_spring_* exhibit mutual cancellation, presenting two symmetric energy barriers ΔE. The former relies on work by the operator when lifting the synchronous ring; the latter depends on potential energy stored within the compression spring. These differing barrier‐crossing mechanisms necessitate two design considerations. First, ΔE and ΔE1 should not be excessively large to minimize the lifting force. Secondly, ΔE1 must exceed the switching potential energy ΔE2, with the surplus accelerating the switching process. Consequently, the spring stiffness *K* is a core parameter, calculated as:

(17) $$K=\frac{Gd^{4}}{8nD^{3}}$$

where *G* is the shear modulus of the spring material, *d* is the spring wire diameter, *D* is the spring's mean coil diameter, and *n* is the number of active coils. It is worth noting that releasing the gripper's constraint on a target, which means transitioning from cage back to parallel, is driven entirely by the stored energy in the system, resulting in a passive, rapid, and driverless mechanism. Figure 5I illustrates the impact of adjusting the wire diameter *d* (1.2–1.8 mm) and mean diameter *D* (12–18 mm) on the spring's potential energy E (ΔE1). The orange curve on the base plane represents the boundary condition (0.24 J). Finally, we select a wire diameter of 1.5 mm and a mean coil diameter of 15 mm for the spring. These values ensure sufficient energy release while avoiding an excessive operational load, achieving an energy‐optimal switching performance (Figure 5J, detailed in Text S9 and Movie S3).

### 2.4 Demonstration of the Gripper Grasping Capability

Building upon the operational scope of conventional soft grippers, our proposed gripper is explicitly designed for extreme scenarios including space, underwater, and disaster environments, aiming to push the boundaries of current applications. In space, robotic systems must confront tumbling debris and uncontrolled tools, while simultaneously manipulating cables and other static objects. Moreover, stringent payload limitations and the high risk of cold welding or mechanical jamming preclude the use of extensive auxiliary equipment. Similarly, underwater operations demand the management of dynamic targets propelled by currents alongside static tasks; however, the waterproofing reliability and prohibitive costs associated with the frequent plugging and unplugging of tool‐changing interfaces are fundamentally unmanageable. Consequently, in such highly unstructured environments governed by severe system‐level constraints, the coexistence of these inherently conflicting functions within a single gripper is not a luxury but an absolute necessity. The system must concurrently possess the capabilities for dynamic target interception and static precision manipulation.

**FIGURE 6:**
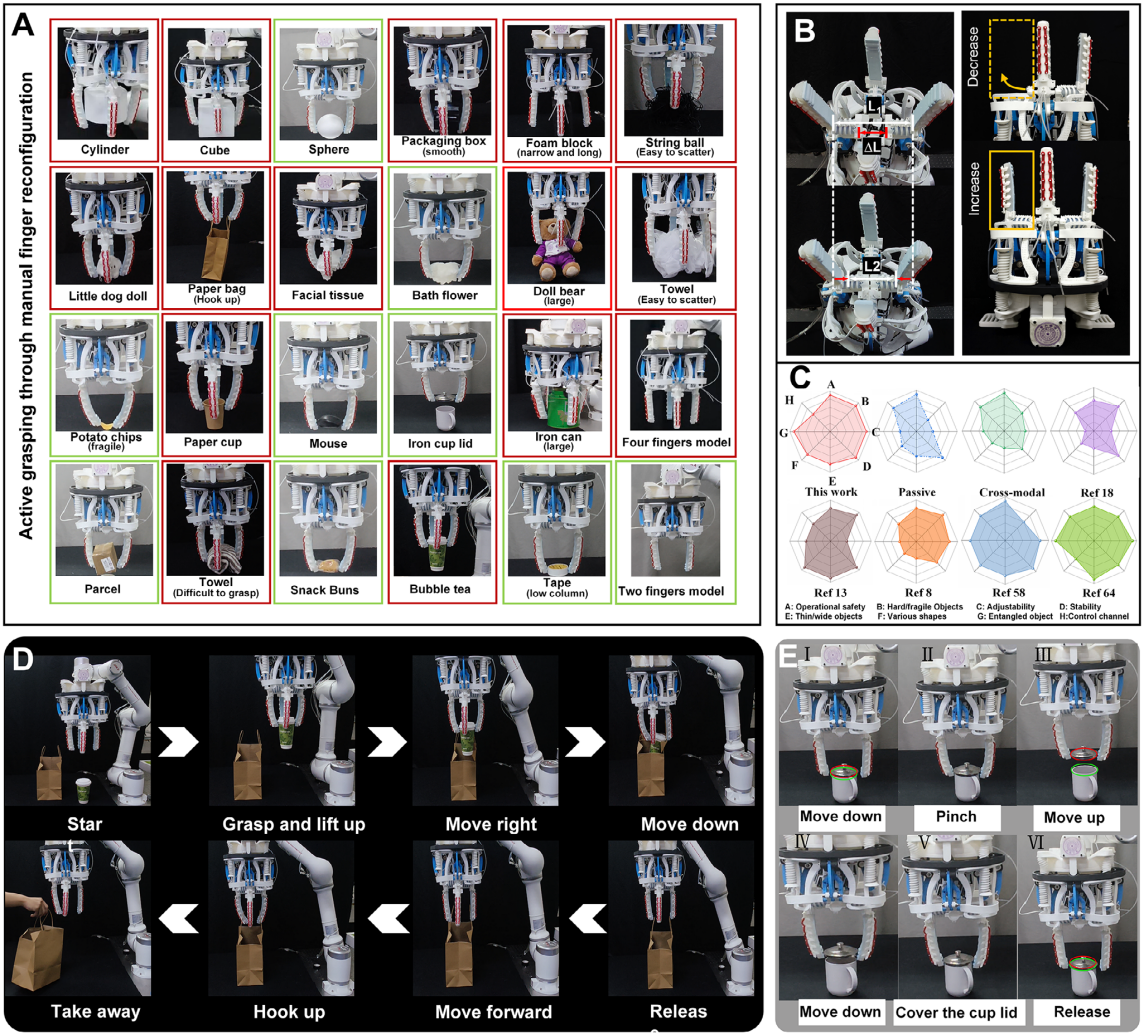
Demonstrations and applications in parallel configuration. (A) The paralleled gripper (green border means two fingers configuration, red border means four fingers configuration) grasps various static targets. (B) Left: images of finger spacing adjustment configurations; right: modular attachment and detachment of fingers. (C) Performance comparison of our gripper with representative soft grippers, including passive grippers, cross‐modal grippers, and other active soft grippers. (D) The gripper performs a trash‐packing task. (E) The gripper picks up and places a metal cup lid with a brim.

To rigorously validate the gripper's application potential under practical experimental conditions, our testing framework is divided into two primary phases: the active grasping of static targets and the dynamic capture of uncooperative targets. We select a broad spectrum of objects and apply diverse parameters under strict conditions. First, in the parallel configuration, we perform active grasping of static targets; the results are shown in Figure 6A. The parallel configuration enables modular attachment, fingers detachment and adjustment of finger spacing (Figure 6B), which expands the size of the captured object and reduces the requirements of redundant drive.

As shown in Figure 6A, the gripper can grasp cylinders, spheres, prisms, and other irregular objects, demonstrating good shape adaptability. In terms of material strength, it can grasp fragile potato chips, soft plush toys, flimsy paper cups, as well as rigid and smooth metal cans. The two‐finger configuration is suited to narrow, lightweight items such as a small bread roll, a mini computer mouse, a potato chip, while four fingers accommodate larger objects. The finger spacing can be adjusted according to object size to avoid the risk caused by over‐pressure. Conventional grippers often fail to grasp objects that are prone to dispersion, but our gripper can stably pick up and release items like a wire mesh, a bundle of string, or a towel by its excellent envelopment. The protruding fingertips design also enables lifting objects such as paper bags and cup lids with brims.

To benchmark the static grasp performance, we compared our gripper to various existing grippers (detailed in Figure 6C). The red solid line represents our gripper, the blue solid line represents the passive grippers, and the green solid line represents the cross‐modal grippers, all the three above are adept at passive grasping. The results show that the latter two grippers suffer noticeable performance degradation when attempting active grasps, which is greatly mitigated in our design. Furthermore, we compare our gripper to some outstanding representatives of active soft hands (dashed lines in Figure 6C). The results indicate that the performance of our gripper is comparable to that of specialized, single‐purpose designs [2, 8, 13, 17, 18, 31, 58, 63, 64]. It should be emphasized that none of those active grippers are capable to capture dynamic targets.

We also conduct two practical task demonstrations (Figure 6D,E, Movie S4). In the first task, the gripper gathers waste and deposits it into a paper bag, then immediately hooks the bag with two fingers and finally lifts it up to a user. In the second task, the gripper pinches and places iron cup lid. Notably, the gripper only has a very small contact area on the smooth, narrow rim of the lid. Thanks to the optimized fingertip force and the protruding structure, the gripper achieves a stable pinch without incidents such as tilting or dropping the lid, firmly placing the lid on the cup.

**FIGURE 7:**
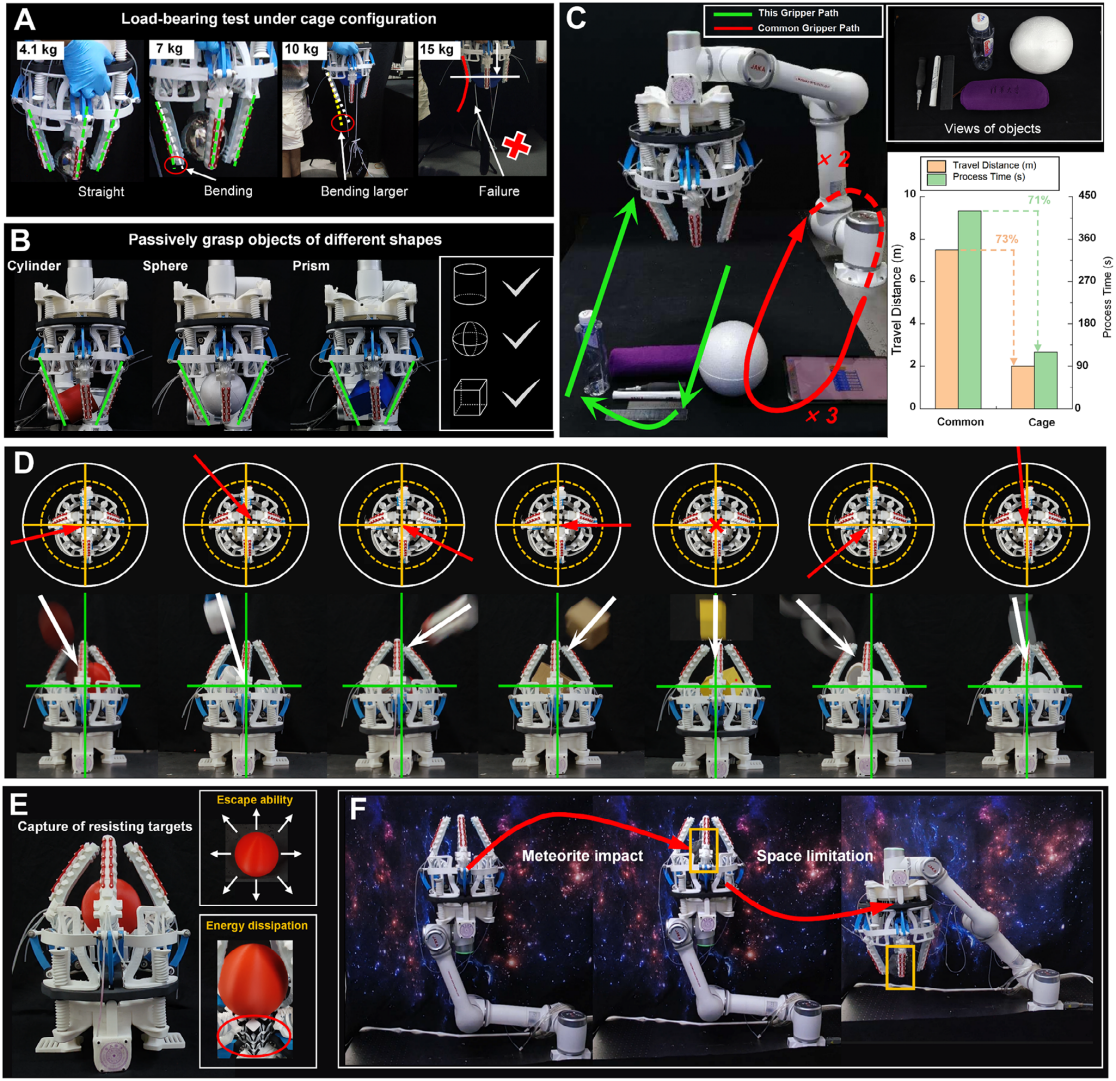
Gripper performance in cage configuration. (A) Load‐bearing capability of the gripper in cage mode. (B) The gripper can lock objects of various shapes, showing high tolerance to shape. (C) Production line simulation shows the comparison of travel distance and operation time between the proposed gripper and a conventional gripper. (D) The gripper passively captures various dynamic targets; the targets vary in size, shape, weight, as well as motion direction and speed. (E) A red ball with omnidirectional mobility remains stably trapped by the gripper. (F) The gripper simulates the capture of an uncooperative space object.

In the cage configuration, the gripper achieves a soft constraint mechanism that is difficult for common passive grippers to realize. Figure 7A shows a load test, when enclosing 4.1 and 7 kg steel balls, the soft finger's profile (green dashed line) deviates slightly from the initial profile (white dashed line), indicating a stable constraint. When lifting a 10 kg barbell plate, the profile (yellow dashed line) deviates by about 20°, which is considered to have reached the safe limit. This weight is 830% of the weight of gripper, demonstrating a remarkable load capacity. At a 15 kg load, Severe deformation occurs in the soft fingers, leading to clear structural failure.

The soft constraint mechanism in the caging gripper also exhibits high tolerance to object shape (Figure 7B, Movie S5), enabling adaptation to various object geometries without complex pre‐planning while maintaining stable locking without requiring sustained energy input.

We conduct an assembly line demonstration in a lab setting (Figure 7C, Movie S5). In this test, after a signal from operator B, operator A needs to transfer over a tool, a workpiece, and a bottle of water. Through the cage mode's spatial allowance, the gripper can load and transport multiple objects in one go. Upon reaching operator B, a light press of the synchronous ring releases all objects for further processing. In this scenario, the gripper functions as a combined storage and transport robot in the production line. Compared to the conventional process of individually locating, picking, and placing each item, this approach achieves approximately 71% reduction in operation time and 73% reduction in travel distance. As the transfer distance increases in practical tasks, this improvement is expected to become even more pronounced.

In previous studies, the dynamic targets captured by grippers often have relatively uniform characteristics. Having overcome this constraint, our experiments now include targets with diverse physical characteristics (shape, mass, speed, incident angle) typical of uncooperative objects. In Figure 7D, from the front and top views, white and red arrows indicate the wide range of incident directions of various objects. Under these complex conditions, the caging gripper achieves exceptional high‐tolerance capture in both spatial and energetic dimensions through a soft constraint strategy, without requiring precise matching and planning for multidimensional parameter variations (see Movie S5). Once a target collided and entered the enclosure, the gripper rapidly imposed a stable spatial constraint. Then, during the collision with the metamaterial palm, nearly all energy of the target is absorbed and dissipated inside the gripper. Experiments document an absence of both rebound phenomena and severe impact. In the first experiment, a fast red ball attempts to roll away after impact, but it is reliably blocked and retained. This demonstrates that the gripper can stably confine objects larger than the gaps between fingers. Furthermore, the gripper can capture targets regardless of its own direction (see Movie S5). For targets capable of active escape, this strategy greatly reduces the intense reactive forces that gripping would induce. In Figure 7E, despite its omnidirectional escape capability, the red ball remains securely confined, exhibiting only vertical bouncing on the palm. The red ball seems to have fallen into a dual trap of energy and space (see Movie S5). Although the gripper exhibits high tolerance to various physical and kinematic parameters of dynamic targets, successful capture is subject to specific size boundaries. Notably, the dimensions of the target object must exceed the 6 cm maximum gap inherent to the cage‐like configuration, yet remain within the 20 cm maximum accessible opening.

Finally, we mount the gripper on a robotic arm to simulate high‐tolerance capture of an uncooperative target in space (Figure 7F, Movie S5). In this experiment, a 2 kg meteorite is launched at high speed from an unknown direction to impact the gripper. The gripper reacts instantaneously and completes the capture, then reorients and smoothly transports the object to a designated location. The meteorite is securely retained by the gripper throughout the entire process, highlighting the promise of our gripper in reliably capturing uncooperative object.

## 3 Conclusion

How to define constitutes “soft” has always been a central question in soft robotics [3, 60, 61, 62]. For soft grippers, it mainly manifests as the high tolerance, which is provided by soft materials and soft contact mechanisms during grasping, resulting in compliant, dexterous, and safe interactions across scales. However, it is worth noting to note that this high tolerance dividend exists primarily for static target grasping; soft grippers often underperform in dynamic capture. This is because they resist rather than utilize interaction forces and compliance, leading to trade‐offs and compromises on design.

In this study, we dexterously propose a soft gripper design that uses dual‐mode morphology to decouple cross‐modal grasping. The gripper seamlessly accommodates both active and passive modes in two morphological states, which are further mapped to soft contact and soft constraint mechanisms, respectively. Driven by this strategy, our soft finger has composite active–passive properties through an exoskeleton–tendon structure. It reconciles the conflicts between soft and rigid, fast and slow. Compared to traditional rigid palms, the biphasic metamaterial palm achieves superior energy absorption and a broader handling range. Under this top‐level coordination of the morphological mechanism, the gripper enhances bidirectional grasping capability for both static and dynamic targets.

Compared to current soft grippers, our cross‐modal gripper can actively grasp static objects with complex features in the parallel mode, and also can store and transfer multiple heterogeneous objects. This functionality allows integration with industrial robotic arms to partially replace the mobile robot in a production line, which can transfer multiple objects. In the cage configuration, the soft constraint mechanism first lets the fingers achieve high tolerance to dynamic targets (shape, size, incident angle, speed) through over‐space constraint, then coordinates with the palm to handle the difficult problem of impact energy dissipation. Both aspects are indispensable for broadening the scope of dynamic capture, indicating great potential for applications like space debris capture and handling of uncooperative targets.

Despite numerous valuable features of our gripper, there are still limitations that can be further optimized. The current morphological switching design emphasizes precise and reliable configuration. In the future, the system could support remote operation and automated mode switching with the motor [18, 57]. Introducing smart responsive materials into the palm and tendons allows for in‐situ tunability [45, 46, 47, 51, 52]. Although the current prototype is relatively bulky and complex, this hardware intricacy strategically reduces dependence on high‐frequency active control, expensive precision sensors for pose estimation, and computationally demanding predictive algorithms. This trade‐off significantly increases functional density and minimizes software overhead. With future advancements in manufacturing technologies, we foresee further structural simplification to enhance practical deployment, despite the anticipated surge in associated costs.

Overall, we present a generalized design strategy that reconciles key conflicts. The work proposes a generalized strategy that employs closed and open geometric configurations to expand and elevate both operational scenarios and performance boundaries. This strategy can be readily extended to other morphological forms. Beyond encompassing conventional applications such as postal logistics and industrial manufacturing, it unlocks new opportunities for extreme environment operations, including marine, disaster response, and space missions. Notably, it successfully overcomes the energetic, spatiotemporal, and control constraints across distinct operational modalities within a single soft robotic system. As progress in tactile sensing and smart materials continues, this solution holds promise for a revolutionary breakthrough in soft grippers.

## 4 Experimental Section

### 4.1 Materials and Fabrication

The fabrication process of the gripper was illustrated in Figure S1. The deformable layers of the grasping module and lateral bending module were molded from Dragon Skin 30 silicone (Smooth‐On Inc.), using inner and outer mold components. The limiting layer was molded from Mold Star 30 and Smooth‐Sil 950 (Smooth‐On Inc.). In each case, silicone Part A and Part B were mixed 1:1 by weight, stirred thoroughly, then degassed in a vacuum chamber to remove bubbles. The degassed silicone was injected into 3D‐printed molds through a feed port using a syringe; after curing at room temperature for 12 h, the part was demolded. Curing was performed in a temperature‐controlled dry box (Zhicheng Co., China). The constraint sheath, rigid inserts, and the synchronous ring and locking mechanism in the switching frame were printed from Vero opaque photopolymer using Stratasys J826 Prime and J850 Prime PolyJet 3D printers. Silicone bonding of the soft finger components was done using Wacker (Germany) Elastosil E43 adhesive and Smooth‐On Sil‐Poxy. The pneumatic actuator tubing is a silicone hose, and the supply airlines are semi‐rigid pneumatic tubes (Soft Touch Co., Ltd., China). Other rigid structural parts were fabricated in ABS plastic. A flexible intercept net used in early prototypes was printed from TPU 95A filament using a Bambu Lab X1‐Carbon printer. The metamaterial palm's lattice was printed using UltiMaker Tough PLA and TPU 95A in a dual‐material UltiMaker S7 Pro Bundle printer (UltiMaker, Netherlands).

### 4.2 Characterization Experiments and Modeling Analysis

For the soft finger simulations: the lateral bending module's deformation under pressurization was analyzed using Abaqus (Dassault Systèmes) to predict the bending angle for different chamber designs (axial vs. 30° angled). The post‐impact bending dynamics of the grasping module were analyzed using Python, with high‐speed video captured by an NPX‐GS6500UM camera for validation. The finger's anisotropic output force was measured using a manual screw‐driven test stand (HLA) equipped with a push–pull force gauge (Handpi, China).

For the metamaterial structure: quasi‐static compression tests of the unit cell were performed on a microcomputer‐controlled universal testing machine (WDW‐5, Lexpert Instruments, China) to obtain the force–displacement curves. Python was used to simulate and fit the response curves. The rebound suppression tests employed a standard baseball dropped through a transparent acrylic tube from a controlled height; rebound was recorded with the NPX‐GS6500UM high‐speed camera. For the morphology switching experiments, a scaled model with a Realme front camera linked to a PC was used to capture and analyse the motion in real time. The kinematic simulation of the entire switching process and the energy analysis were conducted in a Python environment (Pycharm).

### 4.3 Grasping Experiments

The soft gripper prototype was mounted to a UR3 robotic arm (Universal Robots, via JAKA Robotics, China) for grasping experiments. The pneumatic control system for the soft fingers was a customized extension of the SoftTouch system (Soft Touch Co., Ltd., China). The fingers were driven by an Arduino‐controlled pneumatic board, and a LabVIEW program was used for high‐level control and data acquisition at 1 kHz. The arm executed pre‐programmed motions or tele‐operated commands depending on the test (e.g., picking, placing, or intercept trajectories).

### 4.4 Data Analysis

Unless otherwise noted, all experiments were repeated multiple times to ensure data reliability. Data analysis was performed using Origin 2022 (OriginLab), LabVIEW (National Instruments), and Python scripts. All quantitative comparisons (e.g., force measurements, success rates, rebound heights) use mean values from at least 3 trials, with error bars representing the range or standard deviation as appropriate.

## Funding

National Natural Science Foundation of China (General Program) grant 52571385, the Ocean Decade International Cooperation Center (ODCC) grant GHZZ3702840002024020000026, Fujian Ocean Innovation Center grant No. 25FV0CZJ11, the Open Fund of State Key Laboratory of Deep‐sea Manned Vehicles grant No. 2025SKLDMV07, the Shenzhen Science and Technology Program grant Nos. WDZC20231128114452001 and JCYJ20240813112107010, and Shenzhen Key Laboratory of Advanced Technology for Marine Ecology grant No. ZDSYS20230626091459009.

## Conflicts of Interest

The authors declare no conflicts of interest.

## Supporting information




**Supporting File 1**: advs75073‐sup‐0001‐SuppMat.pdf.

**Supporting File 2**: advs75073‐sup‐0002‐MovieS1.mp4.

**Supporting File 3**: advs75073‐sup‐0003‐MovieS2.mp4.

**Supporting File 4**: advs75073‐sup‐0004‐MovieS3.mp4.

**Supporting File 5**: advs75073‐sup‐0005‐MovieS4.mp4.

**Supporting File 6**: advs75073‐sup‐0006‐MovieS5.mp4.

## Data Availability

The data that support the findings of this study are available from the corresponding author upon reasonable request.
