# Supplementary material for: Bioinspired Morphology‐Decoupled Soft Gripper with Enhanced Bidirectional Grasping Capability
Source: Adv Sci (Weinh). 2026 Apr 2;13(34):e75073. doi: 10.1002/advs.75073 (PMC13285120; doi:10.1002/advs.75073)
Supplement: Supplementary file 1 — Supporting File 1: advs75073‐sup‐0001‐SuppMat.pdf. [file ADVS-13-e75073-s006.pdf]

Supporting Information

Bioinspired morphology-decoupled soft gripper with enhanced bidirectional grasping capability

Yedong Huang[1,2], Dazhong Yu[4,5], Baijin Mao[1,2], Fangming Li[1,2], Juntian Qu[1,2,3,]*

[1]Shenzhen International Graduate School, Tsinghua University, Shenzhen 518055, China.
[2]Ocean Decade International Cooperation Center, Qingdao 266000, China.
[3]Fujian Ocean Innovation Center, Xiamen 361102, China.
[4]School of Mechatronical Engineering, Beijing Institute of technology, Beijing 100081, China.
[5]State Key Laboratory of Explosion Science and Safety Protection, School of Mechatronical Engineering, Beijing Institute of technology, Beijing 100081, China

E-mail: juntian.qu@sz.tsinghua.edu.cn

The PDF file includes:

Supplementary Text S1 to S9
Text S1. Design principles of hybrid soft–rigid fingers.
Text S2. Forward large-deformation modeling under impact.
Text S3. Theoretical modeling of the TPU compliant buckling beam.
Text S4. Theoretical analysis of the snap-fit.
Text S5. Theoretical analysis of the overload–tension beam mechanism.
Text S6. Modeling and design for Switching Frame.
Text S7. Gravitational potential energy of the frame.
Text S8. Energy stored in the springs.
Text S9. Spring selection.

Figure S1 to S20
Figure. S1. Structural model of the soft finger
Figure. S2. Vertebral structure and the exoskeletal restraining sheath.
Figure. S3. Finite-element modeling of the lateral-sway module.

Figure. S4. Performance of different cross-sectional finger types when grasping objects of varying sizes.
Figure. S5. Planar deformation analysis of the soft tentacle.
Figure. S6. Flowchart of the iterative solution for planar dynamic capture.
Figure. S7. Calculated deflection angle of the left tentacle after collision..
Figure. S8. Fabrication of the grasping and lateral modules and assembly of the soft finger.
Figure. S9. Theoretical model of the TPU compliant buckling beam.
Figure. S10. Snap-fit interaction.
Figure. S11. The overload–tension beam mechanism.
Figure. S12. Schematic of the gripper switching frame.
Figure. S13. Modeling analysis of the gripper switching frame.
Figure. S14. Four distinct gripper configurations.
Figure. S15. Surface responses of the finger rotation angle and the change in fingertip spacing as functions of expansion length and expansion angle.
Figure. S16. Simplified model of the gripper system and its test and data acquisition setup.
Figure. S17. Simplified structural model of the switching frame.
Figure. S18. Effect of design parameters on gripper motion.
Figure. S19. Suitable design parameters of the switching frame.
Figure. S20. Elastic potential energy of the spring.
Figure. S21 Test curve of unit cell
Figure. S22 Test curve of the elastic tendon

Table S1 to S2
Table S1 Material parameters
Table S2. Simulation parameters.

Supplementary Movies:
Movies S1 to S5

Supplementary Text

Text S1. Design principles of hybrid soft–rigid fingers

The soft finger comprises a lateral module and a grasping module (**Figure. S1(a)**). **Figure. S1(b)** depicts the structural composition of the lateral module, and **Figure. S1(c)** shows the grasping module. An exoskeletal restraining sheath surrounds the soft pneumatic actuator. This sheath interferes with bending in the vertical direction, while, in the lateral direction, it produces a phase-angle-dependent stiffness modulation. The maximum compliant sway angle equals the sum of the sway angles between adjacent constraining rings. **Figure. S1(c)** illustrates the four-layer stack of grasping module, from top to bottom.

Vertebrates have a well-developed skeletal system, and the vertebral column shows pronounced structural specialization that combines flexibility with mechanical robustness. One key factor that enables this performance is the central role of the spinous processes and transverse processes in transmitting and distributing loads. The spine features both types of processes. During longitudinal bending, the spinous process is a prominent posterior midline projection on each vertebra. It serves as an attachment site for many back muscles, such as the trapezius, latissimus dorsi, and rhomboids. Through their insertions on the spinous processes these muscles extend, flex, and rotate the spine. When they contract, they help straighten the spine and maintain an upright posture. Contact and mutual abutment among adjacent spinous processes limit excessive motion and enhance spinal stability. The transverse processes are lateral projections from the vertebrae. They provide attachment points for muscles in the lumbar and cervical regions. They contribute little during modest lateral bending, but they act to constrain motion during excessive lateral sway.

This biological architecture inspired the design of the exoskeletal restraining sheath for the soft finger. Our sheath adopts spinous-like and transverse-like features that provide strong longitudinal load-bearing capacity and limit excessive lateral sway of the actuator. Under longitudinal loading, the spinous-like elements undergo mutual compression and interference. When the lateral sway reaches a certain angle, the transverse-like elements compress and interfere. The corresponding load-bearing tests are presented in the main text.

To better understand and improve how the design influences the finger's output performance and other properties, we model the lateral module and the grasping module separately.

We simulated soft pneumatic actuators with oblique chambers and with straight chambers. Material behavior followed the third-order Yeoh hyperelastic model (*11*),

$W = C_{10}\left(I_1 - 3\right) + C_{20}(I_1 - 3)^2 + C_{30}(I_1 - 3)^3$, which captures orientation hardening at moderate and large strains. Tetrahedral meshes were used together with a hybrid formulation suitable for rubber-like materials. In the lateral module, the central constraining layer was Mold Star 30 and the deformable layer was Dragon Skin 30.

In the lateral module, the deformable layer had a radius of 15 mm and a length of 70 mm. The chamber obliquity was 30° and the wall thickness was 1.5 mm. Each deformable layer contained six sector-shaped pneumatic chambers. The central constraining layer was 3 mm thick. As shown in **Figure. S3**, at identical input pressure the oblique-chamber actuator produced substantially greater lateral actuation than the straight-chamber design. This advantage was most evident before the actuator's sway angle approached the chamber obliquity and diminished thereafter, likely because the deviation between the chamber expansion force vector and the actuator axis decreased.

For the grasping module, bending motion is governed by the differential-strain effect between rigid blocks bonded to an inextensible layer and the soft pneumatic chambers. The governing control equations are as follows:

$$l = \left(1 + \varepsilon_{\text{cham}}\right) l_0 = n_{\text{rigid}} l_{\text{rigid}} + n_{\text{cham}}\left(l_{\text{cham}} + \Delta l_{\text{cham}}\right) \tag{S1}$$

$$\theta = \frac{l}{h' + r} == \frac{n_{\text{rigid}} l_{\text{rigid}} + n_{\text{cham}}\left(l_{\text{cham}} + \Delta l_{\text{cham}}\right)}{h' + R} = \frac{\eta_{\text{rigid}} l_0 + \left(1 + \varepsilon_{\text{cham}}\right)\left(1 - \eta_{\text{rigid}}\right) l_0}{h' + r} \tag{S2}$$

Here, l_0 and l denote the initial length and the axially elongated length of the grasping module. $\varepsilon_{\text{cham}}$ is the chambers' axial strain, θ is the bending angle of the grasping module, and h' is the height at which the maximum axial strain occurs. The bending radius r decreases during bending. n_{rigid} and Δn_{rigid} are the numbers of rigid segments and chambers, respectively, and l_{rigid} is the rigid-segment length. l_{cham} and Δl_{cham} are, respectively, the initial length of the chamber at the soft–rigid contact height and its axial elongation at that height. The contact height h' is assumed to remain constant during inflation.

From Equation (S2), bending is primarily driven by the chambers' axial strain and by the rigid-segment length. At equal pressure, $\varepsilon_{\text{cham}}$ increases with n_{rigid}. In addition, because the rigid blocks cap the chamber tops, a larger n_{rigid} channels a greater fraction of pneumatic energy into axial extension of the grasping module. According to Equation. (S1), the bending angle θ is proportional to the chambers' axial strain (*12*). Increasing that strain therefore improves bending. Incorporating rigid segments between adjacent soft chambers increases the overall axial extension.

In addition, the introduction of rigid segments reduces the damping ratio, which improves force transmission and thereby increases the force transmission efficiency. The fingertip force of the grasping module is taken along the vertical direction. Because the soft chambers and rigid segments alternate while the length of the inextensible layer remains fixed, the simplified kinematic chain behaves as a set of revolute joints. The vertical fingertip force equals the sum of the bending moments contributed by each link. The governing equation is as follows:

$$F_{\text{tip}} = \frac{\sum_{i=1}^{n} M_i}{l_0} = \sum_{i=1}^{n} \frac{M_i}{l_{\text{i}} \cos \theta_{i\,i}} \tag{S3}$$

Here, n is the number of simplified links composed of soft chambers and rigid segments, and M_i is the bending moment of the i th link. During inflation, the length of the lever arm depends on the posture of the grasping module. For analysis, we assume the lever arm length is constant. According to Equation (S3), a larger fingertip force requires a larger total bending moment.

For the tapered fingers (our proposed gripper), fingers generate a nonconstant bending as discussed earlier, thus, when grasping a small-diameter object, the root part of the fingers adapt to its shape, and when grasping medium-sized object, the middle section of the fingers adapt to the object shape, and when grasping large object, the tip part of the fingers adapt to the shape of the object and can successfully grasp (*1,13*)(**Figure. S5**).

Based on the above analysis, we focus on three design principles to enhance the grasping module:

(1) The length of the rigid segments is increased by raising the rigid-segment ratio.

(2) The axial strain of the soft chambers is enhanced by optimizing the cross-sectional shape of the chambers.

(3) Fillets are applied at the interface between the soft and rigid materials to improve the fingertip force.

These factors were validated under various linear pressure loading conditions by experiments based on a Mooney–Rivlin hyperelastic model (*2*).

We define the rigid-to-soft-chamber ratio, η_{rigid}, as follows:

$$\eta_{\text{rigid}} = \frac{n_{\text{rigid}} l_{\text{rigid}}}{l_0} = \frac{l_0 - n_{\text{cham}} l_{\text{cham}}}{l_0} \tag{S4}$$

The cross-sectional shape of the chamber is another key design factor for increasing the bending angle at a given pressure. In a simplified model, the interaction between the soft and

rigid components is represented as a single-link system, and the bending moment can be expressed as follows:

$$M_i = l_i \sin\theta_i F_{i,x} + l_i \cos\theta_i F_{i,y} = l_i \sin\theta_i \int \sigma_{i,x} \mathrm{dA}_{\mathrm{yz}} + l_i \cos\theta_i \int \sigma_{i,y} \mathrm{dA}_{\mathrm{xz}} \quad \text{(S5)}$$

Here, σ_i and $\mathrm{d}A$ are the interfacial stress and the contact area between the soft and rigid structures, respectively. $\sigma_{i,x}$ and $\sigma_{i,y}$ denote the components of σ_i along the x and y directions. The interfacial force F_i is decomposed into x and y components, and the bending moment contributed by each link increases with a rounded-edge configuration. The additional bending moment generated by the rounded edge at the soft–rigid interface is given by:

$$M_{i,top} = \int \sigma_{i,y,e} \mathrm{dA}_{\mathrm{xz}} \quad \text{(S6)}$$

The fingertip force can be expressed as:

$$F_{\mathrm{tip}} = \sum_{i=1}^{n} \frac{\int \sigma_{i,y,e} \mathrm{dA}_{\mathrm{xz}} + l_i \sin\theta_i \int \sigma_{i,x} \mathrm{dA}_{\mathrm{yz}} + l_i \cos\theta_i \int \sigma_{i,y} \mathrm{dA}_{\mathrm{xz}}}{l_i \cos\theta_i} \quad \text{(S7)}$$

In the main text (**Figure. 2E**), comparative tests of three grasping-module designs over 0–50 kPa in 5 kPa increments show that the improved designs increase all mechanical output metrics. The introduction of rigid blocks substantially enhanced the normal contact force (fingertip force), the tangential enveloping force (circumferential grip), and the net grasping force. At 50 kPa, the fingertip forces were 0.743, 1.415, and 1.410 N; the circumferential contact forces were 0.432, 1.587, and 0.631 N; and the net grasping forces were 1.867, 2.998, and 2.794 N, respectively.

Text S2. Forward large-deformation modeling under impact

In passive, restrictive capture of moving targets, the degree of inward bending of the grasping module after impact is a key determinant of whether the target can enter the caged space. We therefore analyze the forward-bending process theoretically. Here, forward bending is defined as the bending that occurs after external loading toward the side with lower stiffness, which corresponds to bending toward the center of the gripper. The deformable layer of the grasping module and the tendon are condensed into an equivalent elastic limiting layer, and the crown-like structures are simplified as adjacent rectangular blocks. The root is fixed and the distal end is free, as shown in **Figure. S5** (*4, 5*). In the initial configuration, before collision with the target, the torques produced by mutual compression among the rigid blocks are balanced by the elastic restoring torque of the deformable bending layer, so the system is in static equilibrium. The corresponding balance equation is:

$$\frac{EI}{r_b}\theta_0 = M_F \tag{S8}$$

In the Equation.S8, r_b is the length of the grasping module, EI is the equivalent flexural rigidity, and θ_0 is the distal deflection angle of the grasping module. When the grasping module undergoes forward bending due to impact, the rigid blocks serve only to transmit the impact force from the target and do not contribute to the overall bending compliance. The forward bending of the grasping module can therefore be treated as the deformation of an equivalent elastic bending layer under load. Let the target mass be m_a, and let the initial speed v_{a0} have a component $v_{a0\perp}$ perpendicular to the line joining the impact point and the root. Let the mass of the grasping module be m_b. The collision occurs at a position P measured from the root. The center of mass remains centered along the module, and the module bends with uniform curvature. The center of mass lies at a distance r_s from the root (with initial value r_{s0}), and its initial speed v_{b0} has a component $v_{b0\perp}$ perpendicular to the line from the center of mass to the root.

After the collision, the target has speed v_a, and the center of mass of the grasping module acquires an angular speed ω about the root and a translational speed v_b. The conservation of angular momentum and the conservation of total mechanical energy are written as follows:

$$m_a v_{a0\perp} P + m_b v_{b0\perp} r_{s0} = m_a v_{a\perp} P + m_b v_{b\perp} r_{s0} \tag{S9}$$

$$\frac{1}{2} m_a v_{a0\perp}{}^2 + \frac{1}{2} m_b v_{b0\perp}{}^2 = \frac{1}{2} m_a v_{a\perp}{}^2 + \frac{1}{2} m_b v_{b\perp}{}^2 \tag{S10}$$

Accordingly, the post-impact velocity components in the perpendicular direction are obtained as follows:

$$v_{a\perp} = \frac{\left(m_a P^2 - m_b r_{s0}{}^2\right) v_{a0\perp} + 2 m_b P r_{s0} v_{b0\perp}}{m_a P^2 + m_b r_{s0}{}^2} \tag{S11}$$

$$v_{b\perp} = \frac{\left(m_b r_{s0}{}^2 - m_a P^2\right) v_{b0\perp} + 2 m_a P r_{s0} v_{a0\perp}}{m_a P^2 + m_b r_{s0}{}^2} \tag{S12}$$

The respective post-impact speeds are:

$$v_a = v_{a0} - v_{a0\perp} + v_{a\perp} \tag{S13}$$

$$v_b = v_{b0} - v_{b0\perp} + v_{b\perp} \tag{S14}$$

Where $r_{s0} = \frac{\sqrt{2} r_b}{\theta_0}\sqrt{1-\cos\frac{\theta_0}{2}}$. For a general configuration, replace r_{s0} and θ_0 with r_s and θ.

After the collision, the grasping module, having acquired kinetic energy, continues to move under inertia and undergoes bending deformation. At some instant during this process, the module reaches bending position in **Figure. S5**, where the distal deflection angle is θ. The kinetic energy at that instant is:

$$T = \frac{1}{2} m_b \left(r_s \frac{\dot{\theta}}{4} \right)^2 = \frac{m_b r_b{}^2 \dot{\theta}^2}{16\theta^2}\left(1-\cos\frac{\theta}{2}\right) \tag{S15}$$

The potential energy of the grasping module is:

$$V = M\frac{\theta}{2} = \frac{1}{2}\frac{EI}{r_b}\theta^2 \tag{S16}$$

Because the grasping module exhibits internal damping that dissipates velocity during deformation, with damping coefficient c, the generalized force associated with this nonconservative damping force is:

$$Q_k{}^* = -c\dot{\theta} \tag{S17}$$

Substituting the above quantities into the Lagrange equation yields the equation of motion with θ as the generalized coordinate:

$$\frac{m_b r_b^{'}}{8\theta^2}\left(1-\cos\frac{\theta}{2}\right)\ddot{\theta} + \frac{m_b r_b^{'}}{32\theta^2}\left(\sin\frac{\theta}{2}\right)\dot{\theta}^2 - \frac{m_b r_b^{'}}{8\theta^3}\left(1-\cos\frac{\theta}{2}\right)\dot{\theta}^2 + \frac{EI}{r_b}\theta + c\dot{\theta} = 0 \tag{S18}$$

Treating θ as the generalized coordinate, the first derivatives of the center-of-mass position give the COM velocity components:

$$v_{xb}=\frac{\mathrm{d}x_b}{\mathrm{d}t}=\frac{r_b}{\theta^2}\left(\frac{\theta}{2}\sin\frac{\theta}{2}+\cos\frac{\theta}{2}-1\right)\dot{\theta} \tag{S19}$$

$$v_{yb}=\frac{\mathrm{d}\,y_b}{\mathrm{d}t}=\frac{r_b}{2\theta^2}\left(\theta\cos\frac{\theta}{2}-2\sin\frac{\theta}{2}\right)\dot{\theta} \tag{S20}$$

For the finger design considered here, the specific simulation parameters are in Table S2.

To resolve the motion of the grasping module over the continuous dynamic bending process, we discretize the deformation into n small steps. Within each time increment Δt, the soft tentacle is assumed to undergo uniformly accelerated motion. By computing the kinematic and dynamic variables at each step, the full process of the deformation is obtained. For sufficiently large n, the resulting discrete simulation closely approximates the true continuous process (*4*).

At time $i\left(i\in[0,n-1]\right)$, the target collides with the grasping module. The center-of-mass position of the module is (x_{bi},y_{bi}). From Equation. S13 and S14, the post-impact COM velocity components are v_{xbi} and v_{ybi}. Substituting x_{bi} and y_{bi} can give the distal deflection angle θ_i:

$$\theta_i=\frac{2r_bx_{bi}}{x_{bi}^{\ 2}+y_{bi}^{\ 2}} \tag{S21}$$

Substituting v_{xbi}, v_{ybi}, and θ_i into Eqs.S19 and S20 yields the distal angular velocity:

$$\dot{\theta}_i=\frac{2\theta_i^2v_{xbi}+\theta_i^3v_{ybi}}{2r_b\left(\frac{\theta_i^2}{4}+1\right)\cos\frac{\theta_i}{2}-2r_b} \tag{S22}$$

Substituting θ_i and $\dot{\theta}_i$ into Eq.S18 gives the distal angular acceleration:

$$\ddot{\theta}_i=\frac{\frac{m_br_b^2}{32\theta_i^2}\left(\sin\frac{\theta_i}{2}\right)\dot{\theta}_i^{\,2}-\frac{m_br_b^{\,2}}{8\theta_i^3}\left(1-\cos\frac{\theta_i}{2}\right)\dot{\theta}_i^{\,2}+\frac{EI}{r_b}\theta_i+c\,\dot{\theta}_i}{\frac{m_br_b^{\,2}}{8\theta_i^2}\left(\cos\frac{\theta_i}{2}-1\right)} \tag{S23}$$

At time $i+1$, the deformation angle and its rate are updated as:

$$\theta_{i+1}=\theta_i+\dot{\theta}_i\Delta t+\frac{1}{2}\ddot{\theta}_i\,\Delta t^2 \tag{S24}$$

$$\dot{\theta}_{i+1}=\dot{\theta}_i+\ddot{\theta}_i \tag{S25}$$

Substituting θ_{i+1} and $\dot{\theta}_{i+1}$ into Eq. S18 to S20 yields $\ddot{\theta}_{i+1}$, $x_{b,i+1}$, $y_{b,i+1}$, $v_{xb,i+1}$, and $v_{yb,i+1}$. The same procedure is repeated for time $i+2$ and subsequent steps. Iteration produces the full deformation history. The iterative procedure is summarized in **Figure. S6.**

Because of the caged geometry and the energy-dissipating role of the palm metamaterial within the caged space, collisions between the target and the opposite (right) finger are not considered here. In this case study, the Young's modulus of the soft finger should exceed 25 MPa to avoid excessive sag under self-weight, while it should not be so large as to preclude the deflection needed to admit most targets.

With the elastic tendon(Ecoflex 00-30) used in our soft finger, the maximum deflection angle reaches 113°, which we consider sufficient to admit the vast majority of targets for successful capture (**Figure. S7**). Dynamic capture experiments demonstrate that the designed soft finger meets the gripper's requirements. Because accurate first-principles prediction of the post-impact rebound time would require highly complex modeling and is sensitive to many practical factors, we measured rebound times experimentally in the main text. The results confirm that the elastic tendon has a positive effect on rebound speed. Rapid recovery is also critical for timely enforcement of the spatial constraint. The manufacturing process and steps of the grasping part are shown in **Figure. S8**.

Text S3. Theoretical modeling of the TPU compliant buckling beam

As shown in **Figure S9**, each metamaterial unit cell has thickness *b* and consists of TPU (thermoplastic polyurethane) buckling beams, a PLA(polylactic acid) snap-fit and a supporting frame.

The model is symmetric about beam OB, geometric constraints cause the resultant force to act normal to the buckling beam AOC, with magnitude F_B. Neglecting small perturbations, OA and OC may be treated as mirror-symmetric about OB during buckling. We therefore analyze only the buckling of the side beam OA. The structures examined in metamaterials unit cell are likewise doubly symmetric in geometry and loading, so the discussion is again restricted to the single-sided deformation mechanism.

In the design, the maximum vertical geometric discrepancy δ between the buckling beam and the straight beam is less than 5% of the length *L*. As the geometry closely resembles the buckled shape of a straight beam under load, we approximate it here as a straight beam.

The vertical beams OB and AD are made of PLA, whose stiffness is far greater than that of the TPU used for the buckling beam AOC. The thicknesses t_2 and t_3 of OB and AD are much larger than t_1, so their deformation is negligible. During unit-cell deformation under load, beam OB provides a strong constraint at joint O and prevents its rotation, while beam AD provides a fixed-end constraint at joint A (*6, 7, 9, 10*)F_B denotes the force acting normal to AOC. As shown in **Figure S9 (b)**, F_O is the force applied to the single-sided model beam OA. By structural and loading symmetry, $2F_O = F_B$.

Subject to the boundary conditions, the reaction bending moment M_O and the horizontal and vertical reactions P_O and F_O at joint *O* are in equilibrium. Consequently, the rotation of beam OA at *O* is zero.

$$\frac{M_O L}{EI} + \frac{L^2 P_O \sin\beta}{2EI} - \frac{L^2 F_O \cos\beta}{2EI} = 0 \tag{S26}$$

I is the moment of inertia of beam OA, given by $I = bt_1^3/12$. In addition, from displacement compatibility at point *O*, the axial displacement δ_a and the transverse displacement δ_t satisfy:

$$\delta_t = \delta_a \tan\beta \tag{S27}$$

The axial displacement δ_a and the transverse displacement δ_t are given by:

$$\delta_t = \frac{F_O L \sin\beta + P_O L \cos\beta}{EA} \tag{S28}$$

$$\delta_a = \frac{F_O L^3 \cos\beta + P_O L^3 \sin\beta}{3EI} - \frac{M_O L^2}{2EI} \tag{S29}$$

Here, the beam cross-sectional area is $A=bt_1$. We denote by κ the ratio of the TPU beam width t_1 to its length L. From Equation S28 and Equation S29, M_O and P_O can be expressed in terms of F_O as:

$$M_O = \frac{F_O L}{2\cos\beta + 2\kappa^2 \tan\beta \sin\beta} \tag{S30}$$

$$P_O = \frac{F_O\left(1-\kappa^2\right)\cot\beta}{1+\kappa^2\cot^2\beta} \tag{S31}$$

Furthermore, from the Euler buckling load and the associated geometric relations, the axial compressive force in beam OA is:

$$N = F_O \sin\beta + P_O \cos\beta = \frac{\pi^2 EI}{(\mu l)^2} \tag{S32}$$

Here, μ is the effective length factor, taken as 0.5. From Equation.(S30)-(S32), and by invoking the symmetry of the model, the resultant reaction of the entire compliant beam is:

$$F_B = \frac{8\pi EI}{L^2} \Bigg/ \left[\left(\sin\beta + \frac{\left(1-\kappa^2\right)\cos^2\beta}{\left(1+\kappa^2\cot^2\beta\right)\sin\beta}\right)\right] \tag{S33}$$

From the above, with the structural thickness b held constant, F_B can be tuned by adjusting κ and the second moment of area I of beam AOC. It should be noted that, to avoid geometric interference between the snap-fit and the buckling beam AOC, the length L of OA and the thickness b are fixed by design. Consequently, F_B is controlled solely through the thickness t_1 of the AOC beam.

Text S4. Theoretical analysis of the snap-fit

As shown in **Figure. S10**, the triangular-protrusion snap (T-snap) has an inclination angle θ and geometric lengths a and b. The circular-protrusion beam (C-beam) is a cantilever of length L_P carrying a circular boss of radius R located a distance L_R from its free end, with bending stiffness EI. The vertical separation between the T-snap and the circular protrusion is d. For design purposes, $R+d$ is prescribed as a constant D.

The snap and the circular protrusion are idealised as rigid bodies at contact. Under load, the T-snap moves towards the C-beam and makes contact with the circular protrusion. During contact there are three characteristic positions, m, n and s, as shown in **Figure S10 (b)**. Points m and n mark the onset and cessation of contact, respectively, while s is where the highest point of the T-snap touches the circular protrusion. In practice, a perfectly sharp point does not exist, and this feature can be neglected for the motion of the T-snap. It is, however, noteworthy because it is useful later for decoupling the parameters L_R and R(*3, 6, 8*).

The deformation process is partitioned according to its geometric characteristics:

$$x = \begin{cases} [0, m) \\ [m, L_p - L_R] \\ (L_p - L_R, n] \\ (n, L_p) \end{cases} \tag{S34}$$

where,

$$m = L_p - L_R + (\cot\theta - \cot\theta\cos\theta + \sin\theta)R + d\cot\theta \quad ,$$

$$n = L_p - L_R + a + b + (\cos\theta + \sin\theta\tan\theta - \tan\theta)R - d\tan\theta$$

Under the small-deflection assumption, the vertical displacement Δy of the C-beam due to compressive contact with the T-snap is obtained.

$$\Delta y = \begin{cases} 0, 0 \le x < m \\ (x - m)\tan\theta, m \le x \le L_p - L_R \\ (n - x)\cot\theta, L_p - L_R < x \le n \\ 0, x > n \end{cases} \tag{S35}$$

As shown in **Figure S10 (c)**, within the contact interval between the T-snap and the circular protrusion, the vertical force on the C-beam, F_V:

$$F_v = \begin{cases} 0, 0 \le x < m \\ \dfrac{3\Delta y EI}{\left(L_R + x - m\right)^3}, m \le x < L_P - L_R \\ \dfrac{3\Delta y EI}{\left(L_R + n - x\right)^3}, L_P - L_R < x \le n \\ 0, x > n \end{cases} \tag{S36}$$

Noting that R<<L_R and n-m<<R, the F_V can be approximated as:

$$F_V \approx \frac{3\Delta y EI}{L_R^{\ 3}} \tag{S37}$$

The friction force experienced by the T-snap during its motion is:

$$f = \mu F = \begin{cases} 0, 0 \le x < m \\ \mu F_v \csc\theta, m \le x \le L_p - L_R \\ \mu F_v \sec\theta, L_p - L_R < x \le n \\ 0, x > n \end{cases} \tag{S38}$$

Finally, the interaction force between the T-snap and the C-beam during the displacement, F^{snap}, is:

$$F^{snap} = \begin{cases} 0, 0 \le x < m \\ \dfrac{6\left(x - m\right)EI}{L_R^{\ 3}}\left(\tan\theta + \mu\right), m \le x \le L_p - L_R \\ \dfrac{6\left(n - x\right)EI}{L_R^{\ 3}}\left(\mu - \cot\theta\right), L_p - L_R < x \le n \\ 0, x > n \end{cases} \tag{S39}$$

Bringing the above analysis together and considering tunability, F^{snap} can be treated as a four-variable function of θ, R, d and L_R .Note that, owing to practical manufacturing tolerances, the adjustable range of θ is only 21.92°, and that of d is only 1.5mm. We therefore tune F_{snap} by varying R and L_R. From Equation.(S39), R and L_R are coupled in their influence on both the peak value of F_{snap} and the position at which it occurs. When the T-snap has moved to point s:

$$F_s^{snap} = \frac{6\mu EI\left(a\tan\theta - D\right)}{L_R^3} + \frac{6\mu EIR}{L_R^3} \tag{S40}$$

In the first term, $a\tan\theta$- D is much smaller than R, and may therefore be approximated as:

$$F_s^{snap} \propto \frac{R}{L_R^3} \tag{S41}$$

Text S5. Theoretical analysis of the overload–tension beam mechanism

As shown in **Figure. S11**, the PLA side beam AB_1 has length L_T and second moment of area I, and is inclined to the base by an angle ω. The buckling beam B_1C_1 as length l. When B_1C_1 is at its initial position, i.e. at the symmetric buckled location C_1, any further loading causes bending of the PLA side beam AC_1 and axial extension of the TPU buckling beam B_1C_1. Pulled by , B_1C_1 the end point B_1 of the side beam moves to B_2, and $\angle B_1AB_2 = \alpha$. It is noteworthy that, during this stage of deformation, the flexural deflection of the PLA beam AB_1 is much larger than the tensile extension of the TPU beam B_1C_1. The tensile deformation of B_1C_1 may therefore be neglected.

End displacement of the side beam m:

$$m = L_T \tan\alpha \tag{S42}$$

Here, x denotes the distance by which the end point C_1 of the TPU buckling beam B_1C_1 moves to C_2 under load, h is the length C_1Q; and a is the length AQ. The length AC_2 can thus be expressed as:

$$AC_2 = \sqrt{\left(h-x\right)^2 + a^2} \tag{S43}$$

From Equation. (S42), the vertical force on the PLA side beam, F_V can be expressed as:

$$F_v = \frac{3EI\tan\alpha}{L_T^2} \tag{S44}$$

where α is given by:

$$\alpha = \omega - \arccos\left(\frac{L_T^2 + \left(h-x\right)^2 + a^2 - l^2 + 2L_T a}{2L_T AC_2}\right) \tag{S45}$$

From Equation. (S43)-(S45), F_V may be regarded as a function of five parameters: l, h, L_T, t, ω. In this model, the four parameters l, h, ω and L_T are coupled to those in the models of appendices A and B, and their tunability is limited owing to potential interference. We therefore pre-program the force F_V on the PLA side beam AB_1 by adjusting the thickness t.

Text S6. Modeling and design for Switching Frame

The gripper's bistable switching frame is shown in **Figure. S12**. The assembly is complex and consists of a multi-link system, a synchronous ring, a fixed ring, latching mechanisms, and spring–slider rail mechanisms. Link 1 connects to the soft finger and, through link 2 and link 4, to the synchronous ring; link 3 connects to the fixed ring. The fixed ring and the synchronous ring are coupled by an energy storage and release module that comprises four spring–slider rail subassemblies and two latching subassemblies. The latch pins engage the groove cams located on both sides of the synchronous ring and within the latch housing. The synchronous ring drives the linkage to execute the design-specified switching path between the parallel configuration and the caged configuration, while the energy storage/release module supplies the energy required to establish each configuration.

To meet requirements, namely small tip spacing in the parallel state, large tip spacing in the caged state, and noninterference between the linkage trajectories and the centrally arranged metamaterial palm during the entire switching process, we construct a model of the switching frame. The model enables refined study of position, orientation, and other kinematic variables with respect to a fixed reference. With conventional methods, the kinematics form a complex system with many nonlinear couplings and with a single input, which is the vertical motion of the synchronous ring, and multiple outputs. The links are directly coupled to the moving input element, which is the synchronous ring, and are only weakly coupled to the static element, which is the fixed ring. As a result, it is difficult to obtain a closed form mapping from the practical input to the several outputs. To address this problem, we propose a dual ring decoupling modeling approach (**Figure. S13**).

First, to simplify the coupled mapping among the links, we treat the synchronous ring as fixed. The fixed ring then serves as the input of the system. This change alters the original input output relationship and reduces the previously complex and difficult to decouple kinematic coupling. It allows us to decompose and analyze the system beginning from the intermediate element that interfaces the multibar actuation and the energy input modules.

Next, we select link 2 as the transitional element. The system is partitioned into two subsystems. Subsystem 1 consists of the fixed ring as the input, link 3 and link 2, and the synchronous ring. Subsystem 2 consists of links 2, 1, 3, and 4. At the subsystem level, in subsystem 1 link 2 is taken as the input and link 4 (AB) is taken as the output. In subsystem 2 the slider is taken as the input, link 3 (DC) acts as the transmission element and functions as a connecting link, and link 2 (OC) is taken as the output. At the macroscopic level link 2 is

therefore the output of subsystem 1 and the input of subsystem 2, which makes it the pivotal variable for system analysis.

We then establish Cartesian coordinate frame at the joint between link 2 and the synchronous ring, and we represent each component by a position vector in this frame. In this way the complex analytical model in conventional formulations is converted into two closed vector loop equations.

$$\vec{l_2}+\vec{l_{12}}=\vec{l_5}+\vec{l_4} \tag{S46}$$

$$\vec{l_2}+\vec{l_3}=\vec{l_6} \tag{S47}$$

Let $\vec{l_2}+\vec{l_{12}}=\vec{l_5}+\vec{l_4}$ denote the first vector loop and $\vec{l_2}+\vec{l_3}=\vec{l_6}$ denote the second vector loop. To facilitate solution of the equations, let θ_i be the angle measured counterclockwise from the *x* axis to the link vector $\vec{l_i}$. Using a complex-valued function:

$$l_2e^{i\theta_2}+l_{12}e^{i\theta_{12}}=l_5e^{i\theta_5}+l_4e^{i\theta_4} \tag{S48}$$

$$l_2e^{i\theta_2}+l_3e^{i\theta_3}=l_6e^{i\theta_6} \tag{S49}$$

Finally, by separating the real and imaginary parts, the transformation matrix for the vector loop equations can be written as:

$$\begin{gathered}\vec{l_2}+\vec{l_{12}}=\vec{l_5}+\vec{l_4}\\ \begin{bmatrix} l_2\cos\theta_2 \\ l_2\sin\theta_2 \end{bmatrix}+\begin{bmatrix} l_{12}\cos\theta_{12} \\ l_{12}\sin\theta_{12} \end{bmatrix}=\begin{bmatrix} l_5\cos\theta_5 \\ l_5\sin\theta_5 \end{bmatrix}+\begin{bmatrix} l_4\cos\theta_4 \\ l_4\sin\theta_4 \end{bmatrix}\end{gathered} \tag{S50}$$

$$\begin{gathered}\vec{l_2}+\vec{l_{12}}=\vec{l_5}+\vec{l_4}\\ \begin{bmatrix} l_2\cos\theta_2 \\ l_2\sin\theta_2 \end{bmatrix}+\begin{bmatrix} l_{12}\cos\theta_{12} \\ l_{12}\sin\theta_{12} \end{bmatrix}=\begin{bmatrix} l_5\cos\theta_5 \\ l_5\sin\theta_5 \end{bmatrix}+\begin{bmatrix} l_4\cos\theta_4 \\ l_4\sin\theta_4 \end{bmatrix}\end{gathered} \tag{S51}$$

$$\begin{gathered}\vec{l_2}+\vec{l_3}=\vec{l_6}\\ \begin{bmatrix} l_2\cos\theta_2 \\ l_2\sin\theta_2 \end{bmatrix}+\begin{bmatrix} l_3\cos\theta_3 \\ l_3\sin\theta_3 \end{bmatrix}=\begin{bmatrix} x_1 \\ -H-\Delta x \end{bmatrix}\end{gathered} \tag{S52}$$

$$l_2\cos\theta_2+l_{12}\cos\theta_{12}=l_5\cos\theta_5+l_4\cos\theta_4 \tag{S53}$$

$$l_2\sin\theta_2+l_{12}\sin\theta_{12}=l_5\cos\theta_5+l_4\sin\theta_4 \tag{S54}$$

$$l_2\cos\theta_2+l_3\cos\theta_3=l_6\cos\theta_6 \tag{S55}$$

$$l_2\sin\theta_2+l_3\sin\theta_3=l_6\sin\theta_6 \tag{S56}$$

The first vector loop describes the relative positions among the local links, and the second vector loop relates the motion of the synchronous ring to the global coordinate frame. The two

subsystem equations are coupled through the link vector $\vec{l}_2$. Direct numerical solution of the nonlinear system is often difficult. We therefore introduce specific geometric conditions and construct auxiliary geometric relations to aid the solution. This approach relies on geometric principles to convert the complicated nonlinear algebraic equations into more intuitive spatial position relations, which simplifies the treatment of constraints and the solution procedure. Here we define a time varying auxiliary line AC and a time invariant auxiliary line AF.

Let φ_1 be the angle between the vector $\vec{l}_5$ and the x axis. Let φ_2 be the angle between $\vec{l}_5$ and l_{AC}, and let φ_3 be the angle between l_{AC} and the vector $\vec{l}_4$. By substituting the variables introduced by the two auxiliary lines with the original variables, we obtain:

$$l_5 = \sqrt{\left(x_1 + x_2\right)^2 + H^2} \tag{S57}$$

$$\varphi_1 = \arccos\frac{x_1 + x_2}{l_5} \tag{S58}$$

$$l_{AC} = \sqrt{l_2^2 + l_5^2 - 2l_5 l_2 \cos\left(\theta_2 + \varphi_1\right)} \tag{S59}$$

In subsystem 1, apply the law of cosines in triangles $\triangle OAC$ and $\triangle ABC$ to compute the auxiliary angles φ_2 and φ_3, which represent the angles between the vector $\vec{l}_5$ and l_{AC}, and between l_{AC} and the vector $\vec{l}_4$, respectively.

$$\varphi_2 = \arccos\frac{l_5^2 + l_{AC}^2 - l_2^2}{2l_5 l_{AC}} \tag{S60}$$

$$\varphi_3 = \arccos\frac{l_4^2 + l_{AC}^2 - l_{12}^2}{2l_4 l_{AC}} \tag{S61}$$

$$\theta_4 = \pi - \varphi_1 - \varphi_2 - \varphi_3 \tag{S62}$$

$$\theta_{12} = \arcsin\frac{l_4 \sin\theta_4 - l_2 \sin\theta_2 - H}{l_{12}} \tag{S63}$$

$$\theta_{11} = \theta_{12} - \alpha \tag{S64}$$

In subsystem 2, the solution is obtained as follows:

$$\theta_3 = \arccos\frac{x_1 - l_2 \cos\theta_2}{l_3} \tag{S65}$$

We then determine the coordinates of the grasping endpoint E and adopt them as a single performance metric. This reduces trajectory planning and attitude control for a multi degree of freedom mechanism to a clear, quantifiable, and globally consistent optimization target. The complex coordination among many links is replaced by focusing on the spatial trajectory of

point E .Using vector superposition with the fixed link $\vec{l_5}$ and links $\vec{l_{12}}$, $\vec{l_{11}}$, and $\vec{l_4}$, the coordinates of E can be written as:

$$\begin{aligned}\overrightarrow{OE} &= \vec{l_5} + \vec{l_4} - \vec{l_{12}} + \vec{l_{11}} \\ &= \begin{bmatrix} x_1 + x_2 \\ -H \end{bmatrix} + \begin{bmatrix} l_4 \cos\theta_4 \\ l_4 \sin\theta_4 \end{bmatrix} - \begin{bmatrix} l_{12} \cos\theta_{12} \\ l_{12} \sin\theta_{12} \end{bmatrix} + \begin{bmatrix} l_{11} \cos\theta_{11} \\ l_{11} \sin\theta_{11} \end{bmatrix}\end{aligned} \quad \text{(S66)}$$

Finally, we seek the mapping between the upward displacement of the synchronous ring, which serves as the input of subsystem 1, and the rotation angle of link 2, which serves as the input of subsystem 2:

$$\begin{bmatrix} l_2 \cos\theta_2 \\ l_2 \sin\theta_2 \end{bmatrix} + \begin{bmatrix} l_3 \cos\theta_3 \\ l_3 \sin\theta_3 \end{bmatrix} = \begin{bmatrix} x_1 \\ -H - \Delta x \end{bmatrix} \quad \text{(S67)}$$

Thus:

$$l_2 \cos\theta_2 + l_3 \cos\theta_3 = x_1 \quad \text{(S68)}$$

$$l_2 \sin\theta_2 + l_3 \sin\theta_3 = -H - \Delta x \quad \text{(S69)}$$

From the foregoing equation:

$$\vec{l_2} = \vec{l_5} + \vec{l_4} - \vec{l_{12}} = \vec{l_6} - \vec{l_3} \quad \text{(S70)}$$

The link vector $\vec{l_2}$ serves as the bridge between the two subsystems. It constrains the system motion and ensures a single degree of freedom. Solving for the angle θ_2 of l_2 is therefore the key to decoupling the entire system. From $\vec{l_2}$ the following constraint relations are obtained:

$$l_5 \cos\theta_5 + l_4 \cos\theta_4 - l_{12} \cos\theta_{12} = l_6 \cos\theta_6 - l_3 \cos\theta_3 \quad \text{(S71)}$$

$$l_5 \sin\theta_5 + l_4 \sin\theta_4 - l_{12} \sin\theta_{12} = l_6 \sin\theta_6 - l_3 \sin\theta_3 \quad \text{(S72)}$$

Because the system of equations contains many trigonometric terms, direct application of conventional numerical solvers such as Newton iteration often fails to converge and can yield solutions that conflict with the actual motion. We therefore reformulate the procedure. Before computing a numerical solution for θ_2, we constrain θ_2 to a physically reasonable interval, and then we solve the equations with a gradient descent method to obtain the correct value of θ_2.

From the system geometry, when links $\vec{l_2}$ and $\vec{l_{12}}$ lie on a common straight line, θ_2 attains its minimum value:

$$\theta_2^{(\text{lower})} = \arccos\frac{(l_2 + l_{12})^2 + l_5^2 - l_4^2}{2(l_2 + l_{12})l_5} - \varphi_1 \quad \text{(S73)}$$

When the linkage reaches the configuration in which links $\vec{l}_4$ and $\vec{l}_{12}$ lie on a common straight line, θ_2 attains its maximum value:

$$\theta_2^{(\text{upper})} = \arccos\frac{l_5^2 + l_2^2 - (l_4 + l_{12})^2}{2l_2 l_5} - \varphi_1 \tag{S74}$$

Let the objective function be $f(t)$, where the input is the candidate angle θ_2 (that is, t), and the output is the squared position error:

$$f(\theta_2) = [x(\theta_2) - \Delta x]^2 \tag{S75}$$

$$x(\theta_2) = -(l_2 \sin\theta_2 + l_3 \sin\theta_3 + H) \tag{S76}$$

This equation uses the forward kinematic model to map θ_2 to the endpoint position $x(\theta_2)$. The squared error measures the deviation from the target displacement Δx, which yields a numerical solution for θ_2. The problem is therefore equivalent to the following mathematical optimization:

$$\begin{gathered}\min f(\theta_2) = [x(\theta_2) - \Delta x]^2 \\ \text{s.t.}\,\theta_2^{(\text{lower})} \le \theta_2 \le \theta_2^{(\text{upper})}\end{gathered} \tag{S77}$$

We solve the bounded single variable nonlinear optimization by a gradient descent algorithm to obtain the optimal θ_2. We first choose the initial value $\theta_2^{(0)}$ as a random number in the interval $\left[\theta_2^{(\text{lower})}, \theta_2^{(\text{upper})}\right]$, and we set the step size $\alpha = 1.2$:

$$\nabla f(\theta_2) = 2[x(\theta_2) - \Delta x]\cdot\frac{dx(\theta_2)}{d\theta_2} \tag{S78}$$

Here, $x(\theta_2)$ is obtained from Equation. (29), and $\mathrm{d}x(\theta_2)/\mathrm{d}\theta_2$ is obtained by combining Equation. (65) and (76):

$$x(\theta_2) = -l_2 \sin\theta_2 - l_3\sqrt{l - \frac{(-l_2\cos\theta_2 + x_1)^2}{l_3^2}} - H \tag{S79}$$

The next iterate of θ_2 is then updated along the negative gradient direction:

$$\theta_2^{(k+1)} = \theta_2^{(k)} - \alpha\cdot\nabla f\left(\theta_2^{(k)}\right) \tag{S80}$$

We then check whether $\theta_2^{(k+1)}$ lies within the admissible interval $\left[\theta_2^{(\text{lower})}, \theta_2^{(\text{upper})}\right]$. If it falls outside the interval, a new initial value $\theta_2^{(0)}$ is drawn and the procedure is repeated until the iterative error meets the prescribed threshold:

$$\left|f\left(\theta_2^{(k+1)}\right)\right| < 1\times10^{-6} \tag{S81}$$

At that point the objective function is considered sufficiently close to zero and the endpoint position error has converged to an acceptable tolerance. The current iterate $\theta_2^{(k+1)}$ is taken as the optimal solution, which indicates successful convergence.

By constructing two independent closed vector loop equations, the complex interdependence among links is decomposed into subproblems that can be solved independently. The double vector loop method determines the positional relationships of the linkage using these closures. This simplifies the solution procedure, reduces computational complexity, and provides a systematic analytical framework for mechanisms that contain multiple closed loops.

Because morphological configuration is critical to gripper performance, we carried out a more precise assessment of the design, configuration, and switching trajectory of the gripper. Guided by the theoretical model and the required configurations, we identify four challenging failure modes in morphology switching. As illustrated in **Figure. S14(a)**, the first mismatch occurs when, under pure rotation about a fixed point, the finger reaches the target switching angle but interference occurs at the fingertip. As shown in **Figure. S14(b)**, the second mismatch occurs in the parallel state when the tip spacing is either too large or too small, which prevents optimal grasping of static targets. Under combined translation and rotation, as shown in **Figure. S14(c)**, the gripper may, over a very small upward stroke, exhibit a rotation angle that is either insufficient or excessive. Therefore, to achieve compatible modes and satisfactory grasping performance in both modes, the gripper must execute a more stringent motion that couple translation and rotation, as illustrated in **Figure. S14(d)**.

We further design the feasible parameter set. During the vertical motion of the synchronous ring, the translational travel of the switching frame endpoint and the rotation angle are the key variables that determine whether the required configurations are achieved. Let the translational distance of point E during the two state transition be d, and let the rotation angle of link l_{11} be θ. In the parallel configuration, d should be small to maintain good grasping performance, and in the caged configuration, d should be large to increase tolerance to target variability. When the synchronous ring moves upward, the resulting θ should be sufficient to close the soft fingers. Based on these considerations, d should lie between 55 and 65 mm. In addition, the switching trajectory must not interfere with the palm located at the center.

To match the size of the soft finger, the grasping envelope, and the manufacturing constraints, the lengths and dimensions of several links in the gripper are determined jointly by an optimization model that enforces the above kinematic requirements, the noninterference condition, and manufacturability bounds.

Using a Python based program, we perform motion modeling of the gripper to satisfy the design conditions. The optimization range for the length of l_{12} is $[25\text{mm}, 35\text{mm}]$, and the optimization range for α is $[125\text{rad}, 155\text{rad}]$. The results are shown in **Figure. S15**. After feasible region screening, we start from the highest point identified by numerical analysis, locate three boundary points, and then carry out screening within the rectangular bounding box.

In addition to evaluating only the initial and final configurations, we also build a Python based simulation to examine the switching trajectory of the gripper. We provide visualized tracking in a supporting video and perform a simplified experiment (**Figure. S16**). Because the gripper configuration exhibits rotational symmetry, key links can be extracted to form a scaled simplified prototype, as shown in **Figure. S17**.

We model the switching trajectories of the gripper for different design parameters in Python. As shown in **Figure. S18**, a coordinate frame is defined with the symmetry axis of the simplified model as the y axis, and the central light blue square denotes the palm region. During switching, the endpoint trajectory must not intersect the palm region. The boundary of the palm is given by:

$$\left\{(x, y) \in \mathbb{R}^2 \mid -x_3 \le x \le -x_3 - l, l_2 \sin\theta_2 - l_3 \sin\theta_3 \le y \le l_2 \sin\theta_2 - l_3 \sin\theta_3 + l\right\} \quad \text{(S82)}$$

Here, x_3 denotes the horizontal distance from the palm region to point O, which is a fixed value during the design process. l represents the side length of the palm region, whose projection onto the two-dimensional plane forms a square.

In the simulation, we evaluate failures induced by parameter choices. In panel (a), the link overhang length is too large (45mm), which produces excessive tip spacing during formation of the caged configuration. If the overhang length is shorter (25mm), as in panel (b), the endpoint interferes with the palm region before the caged configuration is reached. The difference between these two cases is only 20mm.

When the overhang angle is too large (170°), the gripper cannot achieve a suitable tip spacing in the caged configuration. When the overhang angle is too small (130°), the tip spacing in the caged configuration becomes too large and the mechanism tends to approach a dead point. The difference in angle between these two cases is 40°.

These results show that the parameters α and l_{12} can trigger multiple mismatch modes within a relatively narrow range. This finding supports the need for a simulation framework

that enables fine grained evaluation. The outcomes of the scaled simplified experiment agree well with the simulation results and confirm the predictive accuracy.

Finally, within the feasible domain we selected the pair l_{12} = 30 mm and $\theta = 150^{\circ}$ for visualization and a scaled simplified experiment (**Figure. S19**). In both the simulation and the scaled experiment, the trajectory of the lateral sway endpoint did not intersect the palm region, and the caged configuration was achieved.

Text S7. Gravitational potential energy of the frame

The base of the gripper is connected to the robot arm. The gravitational potential energy E_p of the gripper during configuration changes equals the sum of the potential energies of the synchronous ring, the links, and the soft finger. For the calculation, the bottom ring located at point D is chosen as the zero reference, and the zero potential energy surface is constructed with respect to this reference.

Here, each link is modeled as a straight member with a uniform mass distribution. The gravitational potential energy of the n th link equals the product of the link mass and the vertical displacement of its center of mass:

$$E_p^{(n)} = m_n g h_n = \rho_{\text{line}} l_n g \Delta h_n \tag{S83}$$

In Equation.(S83), ρ_{line} is the line density of the link material, and Δh_n is the vertical displacement of the link's center of mass.

In the preceding derivations, the gripper system was expressed in a coordinate frame defined with respect to the synchronous ring, with the origin shown in **Figure. S13**. The resulting expressions describe the coordinate relationship from point O to point E. To compute the change in gravitational potential energy during grasping, the origin is shifted from point O to point D.

In the initial state, denoted as state (0), set $\Delta x^{(0)} = 0\text{mm}$. The centers of mass of all links are specified as follows.

The center of mass of link l_{11} is:

$$\frac{1}{2}\left(\begin{bmatrix} l_2\cos\theta_2 \\ l_2\sin\theta_2 \end{bmatrix} + \begin{bmatrix} l_2\cos\theta_2 \\ l_2\sin\theta_2 \end{bmatrix} + \begin{bmatrix} l_{11}\cos\theta_{11} \\ l_{11}\sin\theta_{11} \end{bmatrix}\right) - \left(\begin{bmatrix} l_2\cos\theta_2 \\ l_2\sin\theta_2 \end{bmatrix} + \begin{bmatrix} l_3\cos\theta_3 \\ l_3\sin\theta_3 \end{bmatrix}\right) = \begin{bmatrix} \dfrac{l_{11}\cos\theta_{11}}{2} - l_3\cos\theta_3 \\ \dfrac{l_{11}\sin\theta_{11}}{2} - l_3\sin\theta_3 \end{bmatrix}$$

(S84)

$$h_{11}^{(0)} = \frac{l_{11}\sin\theta_{11}}{2} - l_3\sin\theta_3 \tag{S85}$$

The center of mass of link l_{12} is:

$$\frac{1}{2}\left(\begin{bmatrix} l_2\cos\theta_2 \\ l_2\sin\theta_2 \end{bmatrix} + \begin{bmatrix} l_2\cos\theta_2 \\ l_2\sin\theta_2 \end{bmatrix} + \begin{bmatrix} l_{12}\cos\theta_{12} \\ l_{12}\sin\theta_{12} \end{bmatrix}\right) - \left(\begin{bmatrix} l_2\cos\theta_2 \\ l_2\sin\theta_2 \end{bmatrix} + \begin{bmatrix} l_3\cos\theta_3 \\ l_3\sin\theta_3 \end{bmatrix}\right) = \begin{bmatrix} \dfrac{l_{12}\cos(\theta_{12})}{2} - l_3\cos(\theta_3) \\ \dfrac{l_{12}\sin(\theta_{12})}{2} - l_3\sin(\theta_3) \end{bmatrix}$$

(S86)

$$h_{12}^{(0)} = \frac{l_{12}\sin(\theta_{12})}{2} - l_3\sin(\theta_3) \tag{S87}$$

The center of mass of link l_2 is:

$$\frac{1}{2}\begin{bmatrix} l_2\cos\theta_2 \\ l_2\sin\theta_2 \end{bmatrix} - \left(\begin{bmatrix} l_2\cos\theta_2 \\ l_2\sin\theta_2 \end{bmatrix} + \begin{bmatrix} l_3\cos\theta_3 \\ l_3\sin\theta_3 \end{bmatrix}\right) - \left(\begin{bmatrix} l_2\cos\theta_2 \\ l_2\sin\theta_2 \end{bmatrix} + \begin{bmatrix} l_3\cos\theta_3 \\ l_3\sin\theta_3 \end{bmatrix}\right) = \begin{bmatrix} -\frac{1}{2}l_2\cos\theta_2 - l_3\cos\theta_3 \\ -\frac{1}{2}l_2\sin\theta_2 - l_3\sin\theta_3 \end{bmatrix}$$

(S88)

$$h_2^{(0)} = -\frac{1}{2}l_2\sin\theta_2 - l_3\sin\theta_3 \tag{S89}$$

The center of mass of link l_3 is:

$$\frac{1}{2}\left(\begin{bmatrix} l_2\cos\theta_2 \\ l_2\sin\theta_2 \end{bmatrix} + \begin{bmatrix} l_2\cos\theta_2 \\ l_2\sin\theta_2 \end{bmatrix} + \begin{bmatrix} l_3\cos\theta_3 \\ l_3\sin\theta_3 \end{bmatrix}\right) - \left(\begin{bmatrix} l_2\cos\theta_2 \\ l_2\sin\theta_2 \end{bmatrix} + \begin{bmatrix} l_3\cos\theta_3 \\ l_3\sin\theta_3 \end{bmatrix}\right) = -\frac{1}{2}\begin{bmatrix} l_3\cos\theta_3 \\ l_3\sin\theta_3 \end{bmatrix} \tag{S90}$$

$$h_3^{(0)} = -\frac{1}{2}l_3\sin\theta_3 \tag{S91}$$

The center of mass of link l_4 is:

$$\frac{1}{2}\left(\begin{bmatrix} l_2\cos\theta_2 \\ l_2\sin\theta_2 \end{bmatrix} + \begin{bmatrix} l_{12}\cos\theta_{12} \\ l_{12}\sin\theta_{12} \end{bmatrix} + \begin{bmatrix} l_2\cos\theta_2 \\ l_2\sin\theta_2 \end{bmatrix} + \begin{bmatrix} l_{12}\cos\theta_{12} \\ l_{12}\sin\theta_{12} \end{bmatrix} - \begin{bmatrix} l_4\cos\theta_4 \\ l_4\sin\theta_4 \end{bmatrix}\right) - \left(\begin{bmatrix} l_2\cos\theta_2 \\ l_2\sin\theta_2 \end{bmatrix} + \begin{bmatrix} l_3\cos\theta_3 \\ l_3\sin\theta_3 \end{bmatrix}\right)$$

$$= \begin{bmatrix} l_{12}\cos(\theta_{12}) - l_3\cos(\theta_3) - \frac{l_4\cos(\theta_4)}{2} \\ l_{12}\sin(\theta_{12}) - l_3\sin(\theta_3) - \frac{l_4\sin(\theta_4)}{2} \end{bmatrix}$$

(S92)

$$h_4^{(0)} = l_{12}\sin(\theta_{12}) - l_3\sin(\theta_3) - \frac{l_4\sin(\theta_4)}{2} \tag{S93}$$

Since the soft finger does not change shape during switching, it can be modeled as a point mass F whose position relative to link l_{11} is fixed. Connecting point F to point C defines link l_6. The mass of point F equals the mass of the soft finger. The position of F is:

$$\begin{pmatrix} x \\ y \end{pmatrix} = \begin{bmatrix} l_2\cos\theta_2 \\ l_2\sin\theta_2 \end{bmatrix} + \begin{bmatrix} l_7\cos\theta_7 \\ l_7\sin\theta_7 \end{bmatrix} \tag{S94}$$

$$h_7^{(0)} = l_2\sin\theta_2 + l_7\sin\theta_7 \tag{S95}$$

When the gripper switches to the caged configuration, denoted as state (1), set $\Delta x^{(1)} = 20\text{mm}$. Repeating the above procedure yields $h_n^{(1)}$:

$$\Delta h_n = h_n^{(1)} - h_n^{(0)} \tag{S96}$$

For the synchronous ring, only vertical translation occurs during switching, so the change in its gravitational potential energy is:

$$E_{P,ring} = m_{\text{ring}} g \left(\Delta x^{(1)} - \Delta x^{(0)} \right) \quad \text{(S97)}$$

Where m_{ring} is the mass of the synchronous ring. Therefore, the change in the gripper's gravitational potential energy during the configuration transition is:

$$\Delta E_p = 4 \sum \rho_{\text{line}} l_n g \left(l_{11} \Delta h_{11} + l_{12} \Delta h_{12} + l_2 \Delta h_2 + l_3 \Delta h_3 + l_4 \Delta h_4 \right) + 4 m_7 g \Delta h_7 + m_{\text{ring}} g \Delta x \quad \text{(S98)}$$

The total energy of the system equals the superposition of the gravitational potential energy of the frame and the elastic potential energy of the springs, which reflects the energetic balance during motion. Adding the spring energy to the frame's gravitational energy gives:

$$\begin{aligned} E_{\text{total}} &= E_p + E \\ &= 4 \sum \rho_{\text{line}} l_n g \left(\sum_{i=1}^{2} l_{1n} \Delta h_{1n} + \sum_{i=2}^{4} l_n \Delta h_n \right) + 4 m_7 g \Delta h_7 + m_{\text{ring}} g \Delta x + K\, L\, \Delta x + \frac{K\, \Delta x^2}{2} \end{aligned} \quad \text{(S99)}$$

When Δx varies within $[-20\text{mm}, 20\text{mm}]$, the resulting energy curve is obtained (Fig 5H). The inflection points have specific physical meaning, namely that the mechanism is in an energy optimized state when the rate of change of the total energy is minimal. These locations can be used as ideal positions for grasping. For clarity of comparison, the final state gravitational potential energy is often taken as the zero reference so that the curve more directly shows relative changes.

Text S8. Energy stored in the springs

Springs are key energy conversion components in the gripper system and they provide the driving power for configuration switching. In the structural design of the gripper, one end of each spring is fixed to the bottom ring at point D, and the other end is fixed to the synchronous ring at point O. The elastic potential energy of the spring is obtained from Hooke's law as:

$$E = K\int_{L}^{L+\Delta x} x\,dx = K\,L\,\Delta x + \frac{K\,\Delta x^2}{2} \tag{S100}$$

where L is the initial compression of the spring in the reference configuration, K is the spring stiffness, and Δx is the additional displacement during switching. The stiffness K is determined by the spring's material and geometry, and for a helical compression spring is given by:

$$K = \frac{Gd^4}{8nD^3} \tag{S101}$$

Where G is the shear modulus of the spring material, d is the wire diameter, D is the mean coil diameter, and n is the number of active coils.

Text S9. Spring selection

For a cylindrical helical spring, the stiffness K is given by:

$$K = \frac{Gd^4}{8nD^3} \quad \text{(S102)}$$

Where G is the shear modulus of the spring material(SUS304-WPB steel, G = 76.5×10^9 Pa), d is the wire diameter (m), D is the mean coil diameter (m), and n is the number of active coils $(n = 14)$.When the spring in the gripper mechanism is compressed by $\Delta x = 20\mathrm{mm}$, a parameter sweep over D and d yields the energy distribution shown in Fig. S18.

Typical parameter result. For D = 15 mm and $d = 1.5\mathrm{mm}$, the spring stiffness is $k = 120.743\mathrm{gf\ mm}^{-1}$ and the elastic energy stored in the spring is $E = 0.237\mathrm{J}$.The minimum energy is $\min(E) = 0.072\mathrm{J}\,(D = 17.5\mathrm{mm}, d = 1.25\mathrm{mm})$, and the maximum energy is $\max(E) = 0.758\mathrm{J}\,(D = 12.5\mathrm{mm}, d = 1.75\mathrm{mm})$.

The spring energy increases with wire diameter d and decreases nonlinearly with mean diameter D, consistent with the theoretical relation $E \propto d^4 / D^3$. Balancing energy margin and structural strength, the recommended selection is $D = 15\mathrm{mm}$ and $d = 1.5\mathrm{mm}$. In this case the stored spring energy is moderate, $E = 0.237\mathrm{J}$, and the stiffness matches the change in gravitational potential energy of the gripper system (**Figure**. 5I).

Text S10 Fatigue testing of the relevant components

In our design, ensuring the stability of the hybrid rigid and soft coupling interface within the metamaterial unit cell and guaranteeing the reliability of the elastic tendon are of paramount importance. Therefore, we conduct dedicated experimental evaluations on both the unit cell material interface and the elastic tendon.

For the interface of the unit cell, we select Bambu PLA Basic and Bambu TPU 95A HF for fabrication. The printed components exhibit smooth and flat surfaces with excellent manufacturing quality and fine layer resolution. We utilize a printing infill density of 100%. The boundary between these two materials features a mechanical mortise and tenon interlocking connection, which delivers exceptionally robust connection strength. We secure the upper and lower surfaces of the unit cell onto a force testing machine and subject the structure to a comprehensive compression test comprising 500 complete cycles. Subsequent data analysis reveals that the mechanical properties of the unit cell show no noticeable degradation and maintain excellent structural integrity (**Figure** S21).

The elastic tendon is fabricated from the Ecoflex 00-30 elastomer. This material is exceptionally soft, durable, and elastic. It can stretch to multiple times its original dimensions without tearing and recover its initial shape elastically, boasting a theoretical elongation at break of up to 900%. In our specific experimental evaluations, despite the unique structural geometry of our elastic tendon, its empirical elongation at break remains remarkably high at 500%. During actual operation, the required stretch ratio for our tendon is merely 30% to 40%. This falls drastically below its ultimate tensile limit and utilizes only a tiny fraction of its total capacity. In addition to these ultimate limit evaluations, we supplement our study with cyclic fatigue tests involving 500 continuous stretching cycles. We anchor the elastic tendon within the testing machine clamps for dynamic evaluation. The results confirm that the mechanical performance of the tendon remains virtually unchanged from the beginning to the end of the cyclic experiment (**Figure** S22).

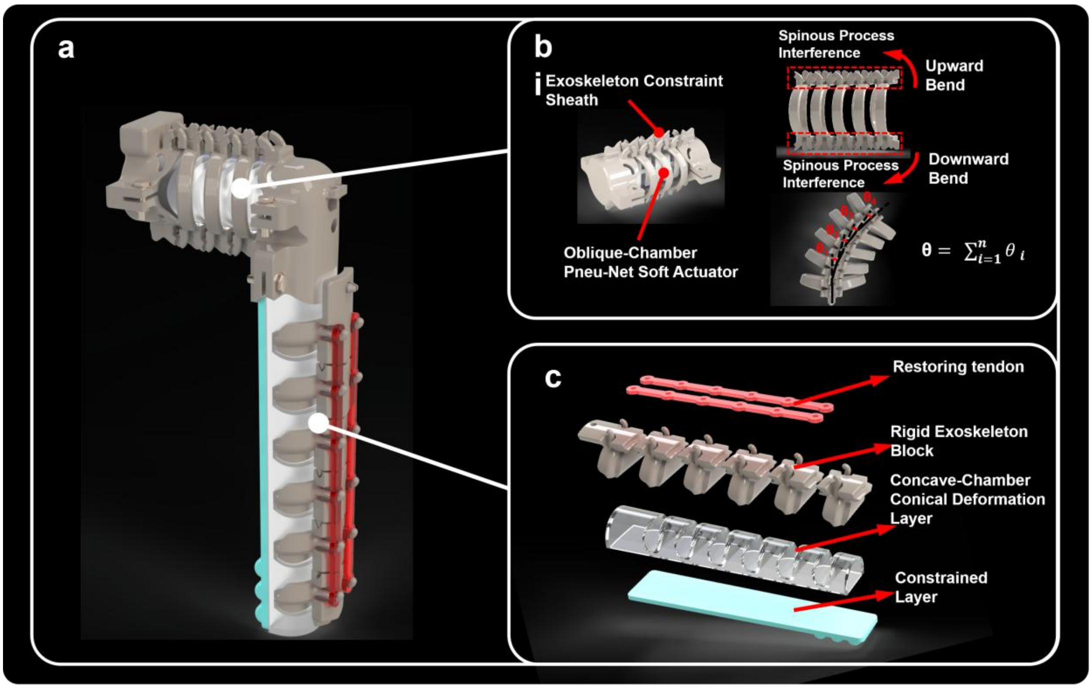

Figure. S1. Structural model of the soft finger. (a) Overview. (b) Exoskeleton constraint sheath details (c) Grasping module. Four-layer stack shown from top to bottom.

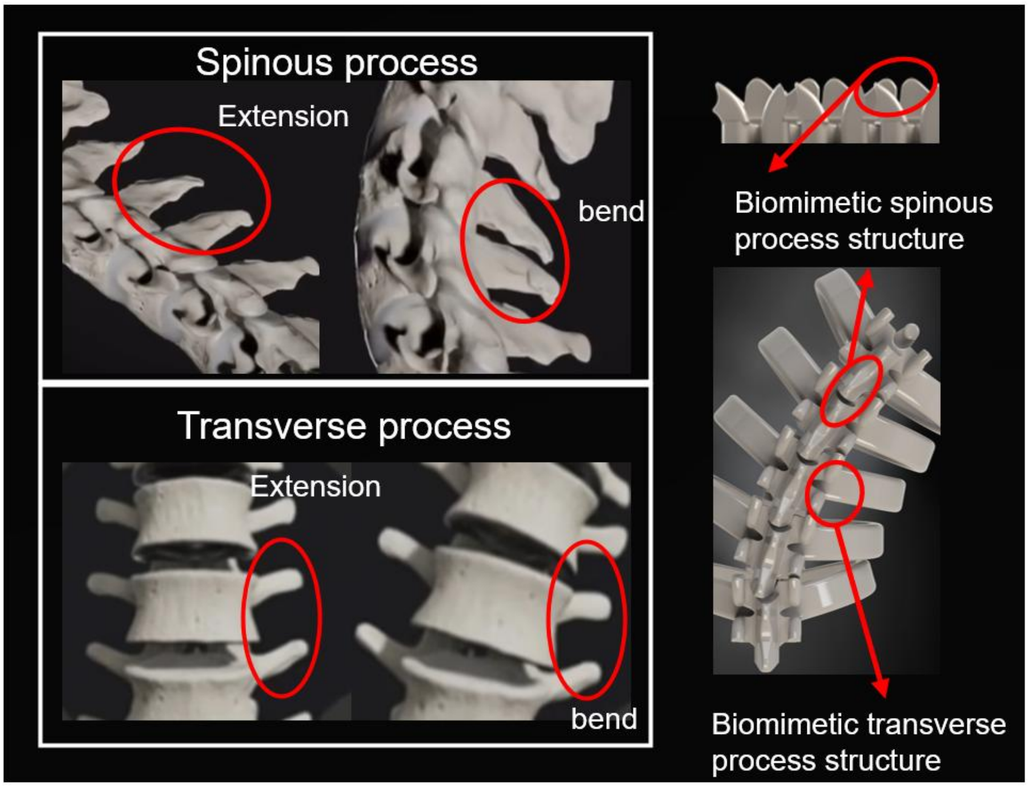

Figure. S2. Vertebral structure and the exoskeletal restraining sheath.

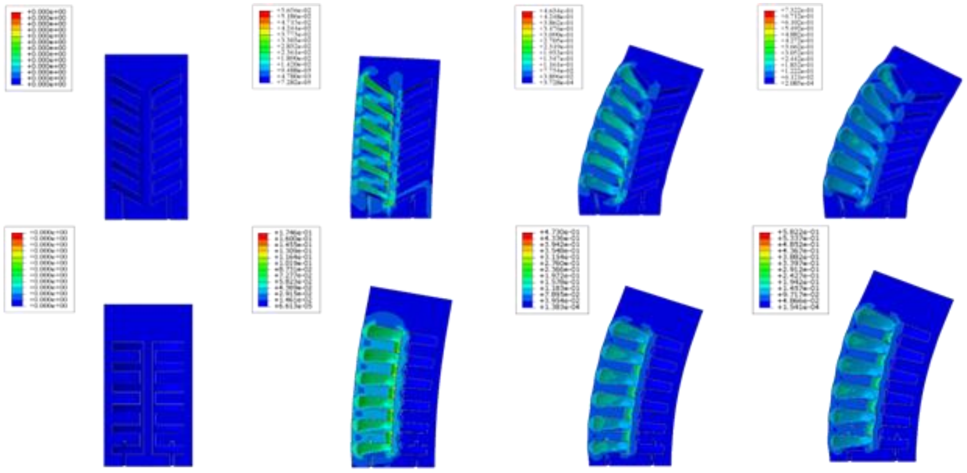

Figure. S3. Finite-element modeling of the lateral-sway module.

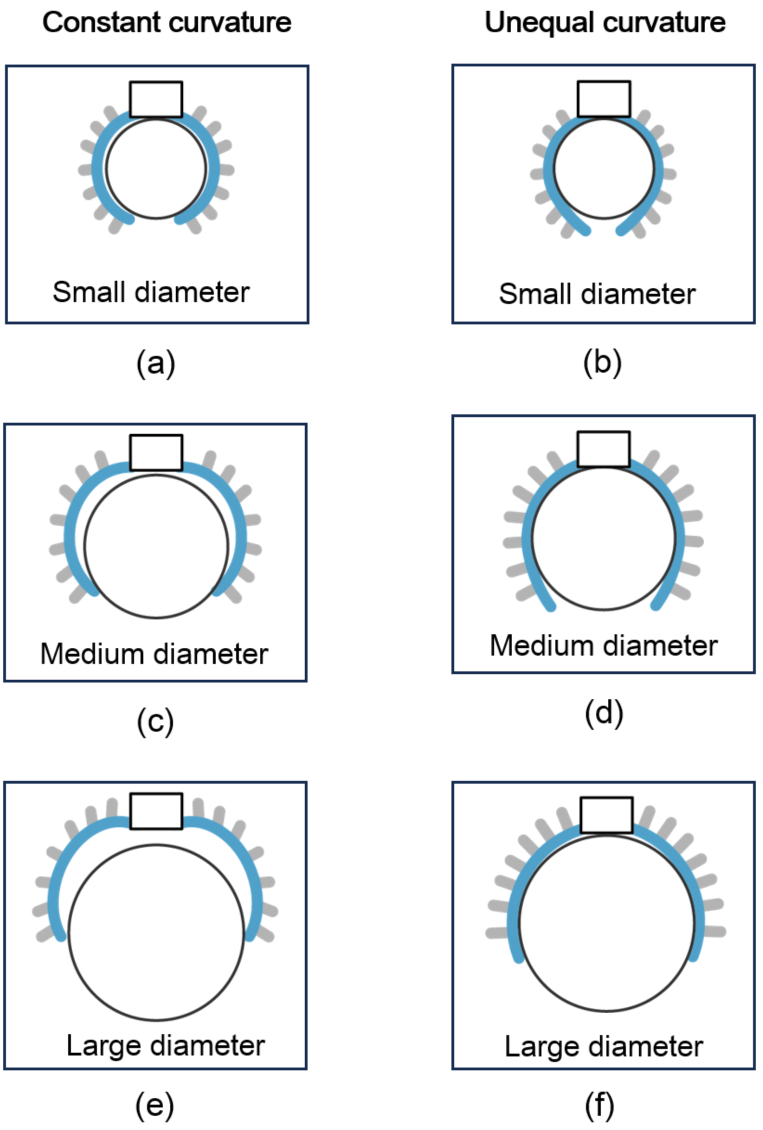

Figure. S4. Performance of different cross-sectional finger types when grasping objects of varying sizes. (a) Constant cross-section fingers gripping a small cylinder. (b) Tapered cross-section fingers gripping a small cylinder. (c) Constant cross-section fingers gripping a medium-sized cylinder. (d) Tapered cross-section fingers gripping a medium-sized cylinder. (e) Constant cross-section fingers gripping a large cylinder. (f) Tapered cross-section fingers gripping a large cylinder. Tapered cross-section fingers demonstrate superior adaptability to the size and shape of objects during grasping.

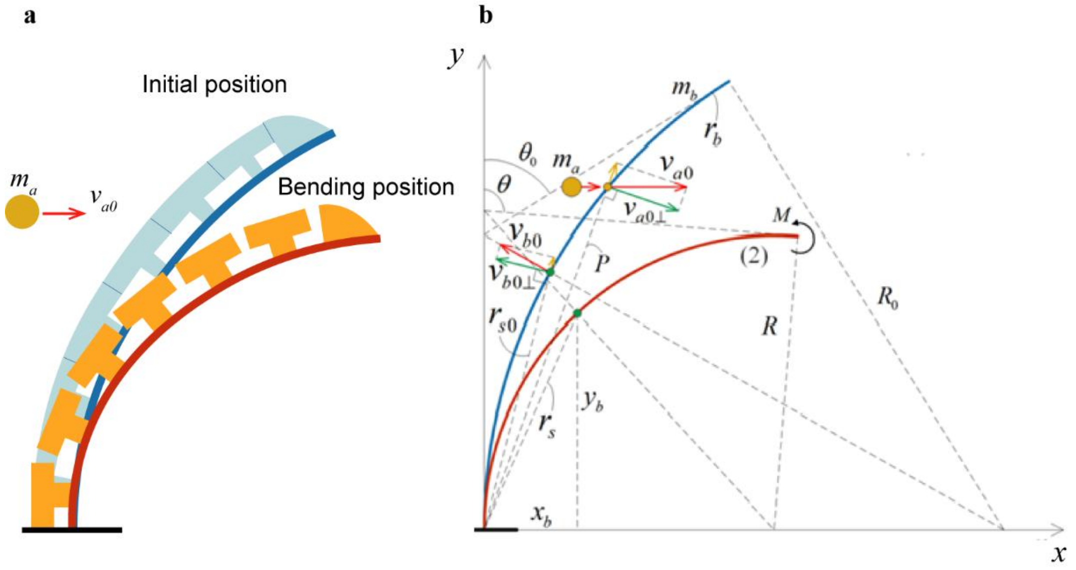

Figure. S5. Planar deformation analysis of the soft tentacle. (a) Simplified model of the grasping module. (b) Simplified geometry of the grasping module.

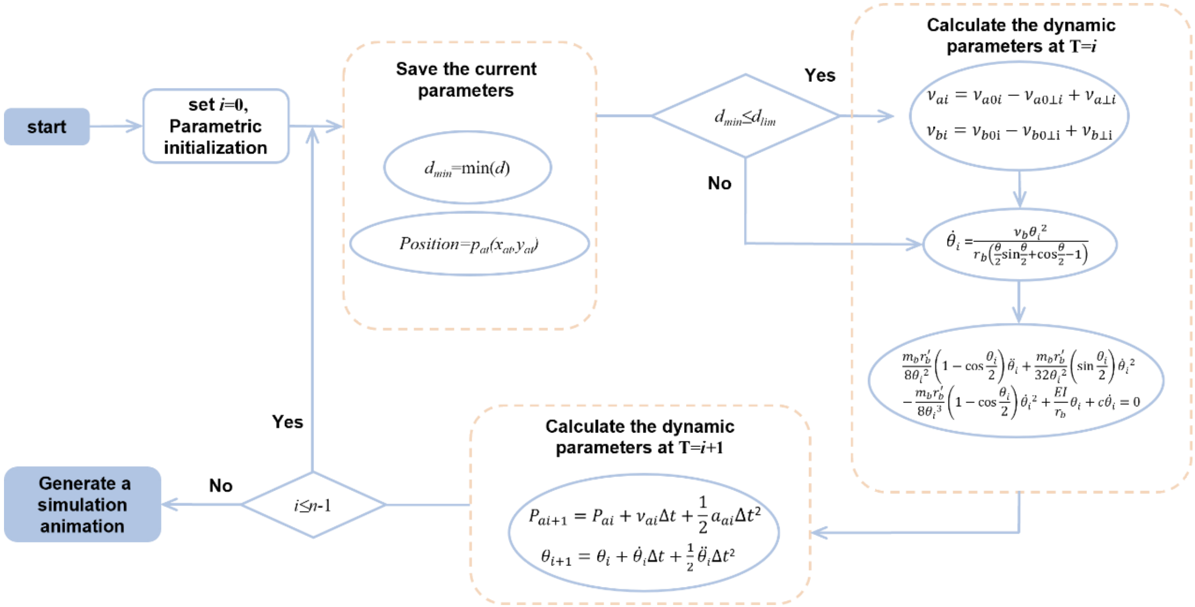

Figure. S6. Flowchart of the iterative solution for planar dynamic capture.

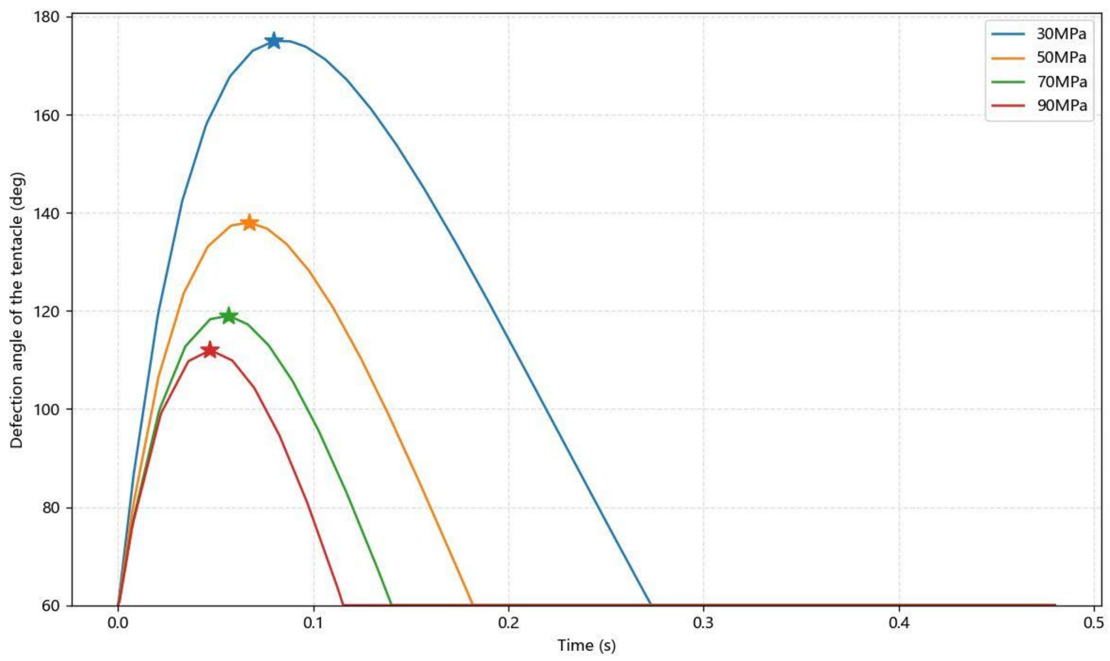

Figure. S7. Calculated deflection angle of the left tentacle after collision..

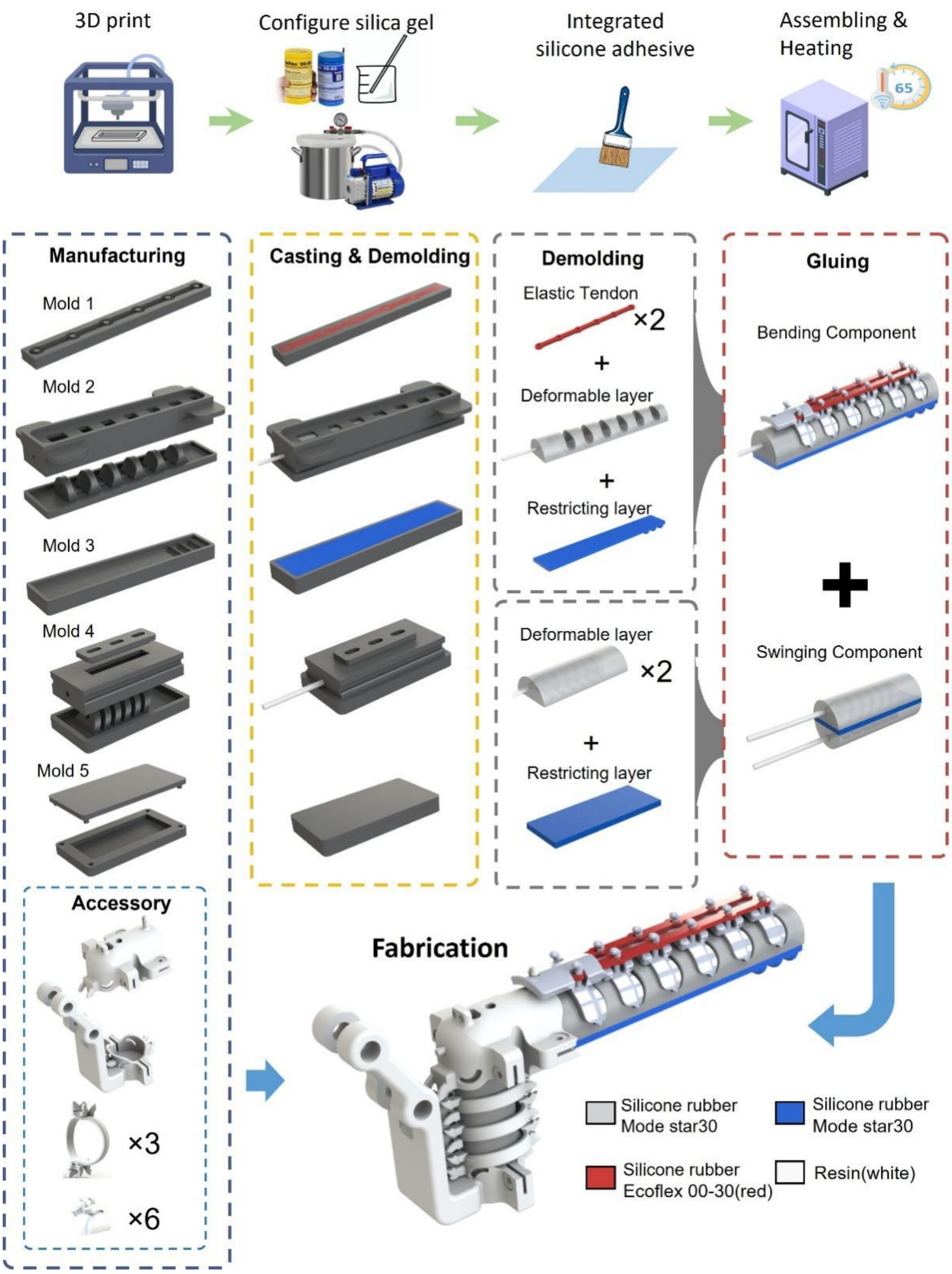

Figure. S8. Fabrication of the grasping and lateral modules and assembly of the soft finger.

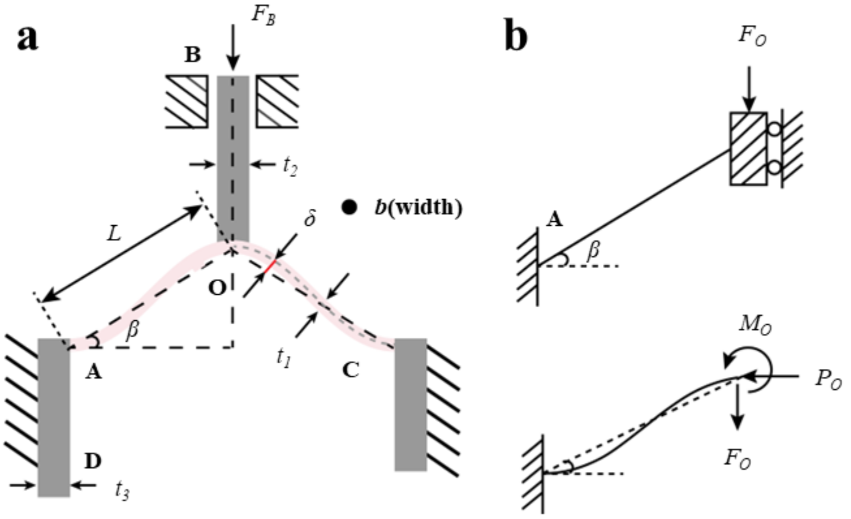

Figure. S9. Theoretical model of the TPU compliant buckling beam. (a) Metamaterial unit cell of thickness b comprising a TPU buckling beam AOC, a PLA snap-fit, and a PLA supporting frame. The geometry is symmetric about beam OB. (b) Single-sided model of beam OA under a resultant force F_O. The PLA beams OB and AD provide a rotational constraint at O and a fixed end at A, respectively.

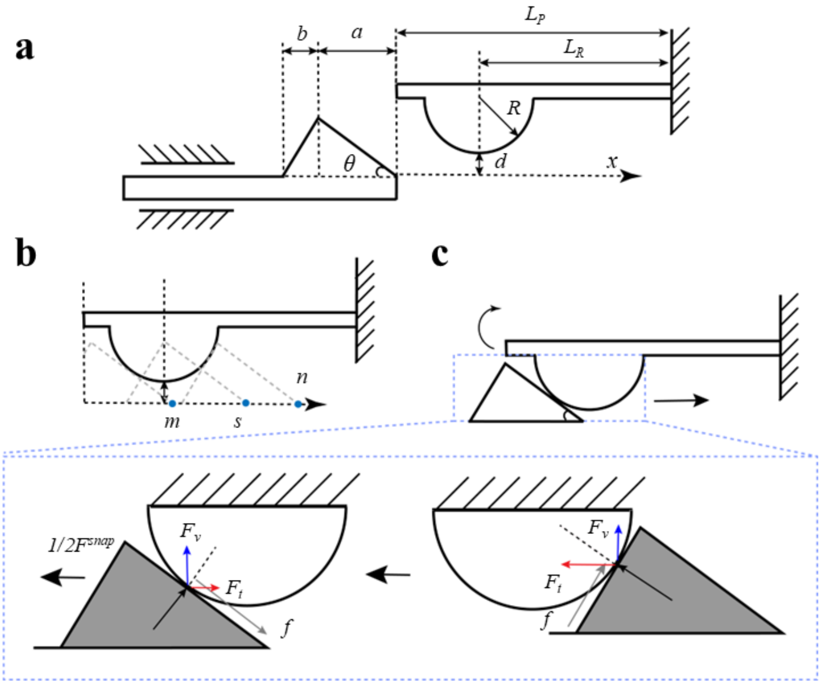

Figure. S10. Snap-fit interaction. (a) Geometry of the triangular-protrusion snap and the circular-protrusion cantileve.(b) Characteristic contact positions (c) Vertical force on the C-beam during contact.

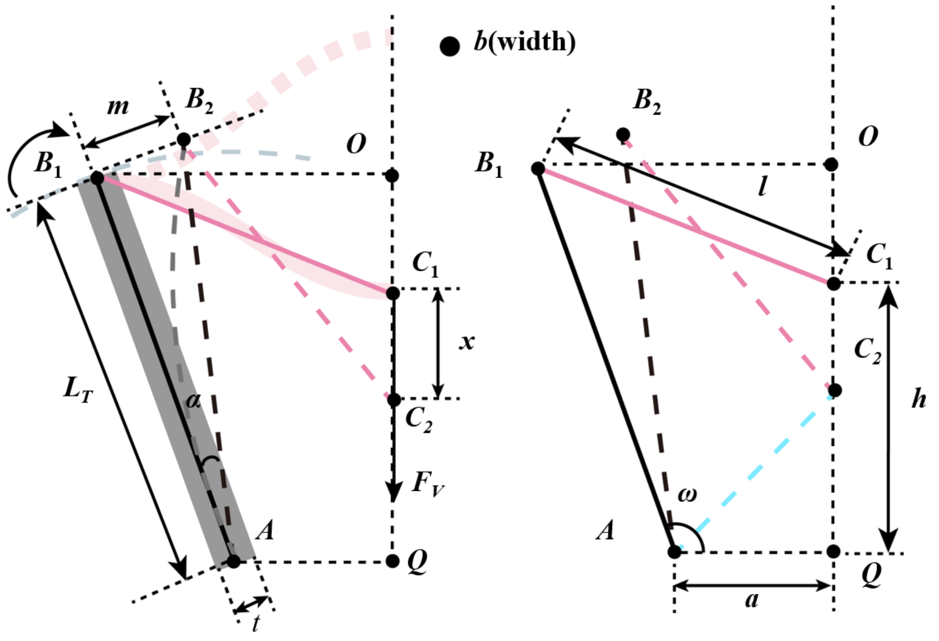

Figure. S11. The overload–tension beam mechanism.

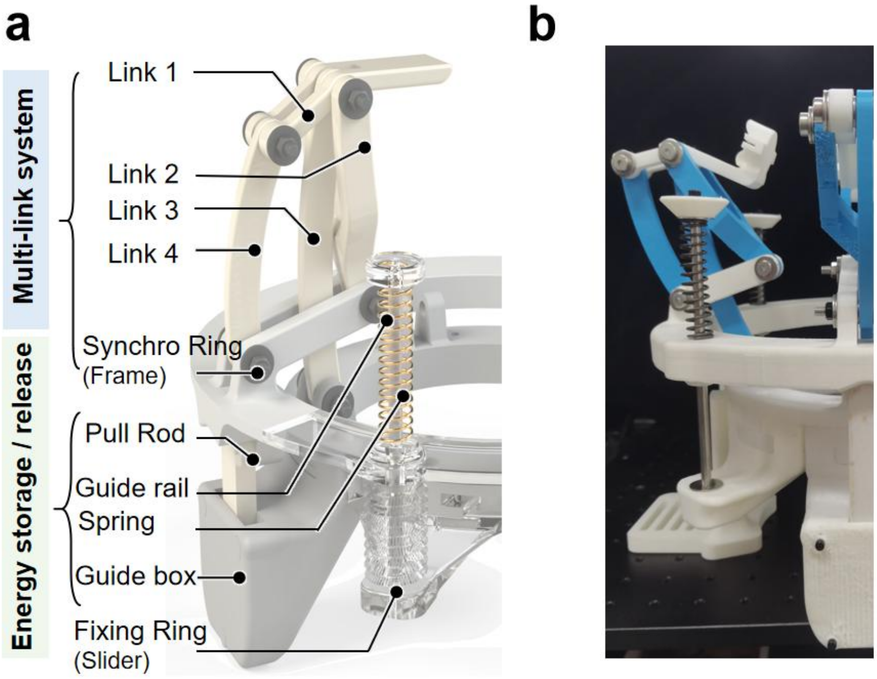

Figure. S12. Schematic of the gripper switching frame. (a) Structural model of the switching frame. (b) Photograph of the switching frame prototype.

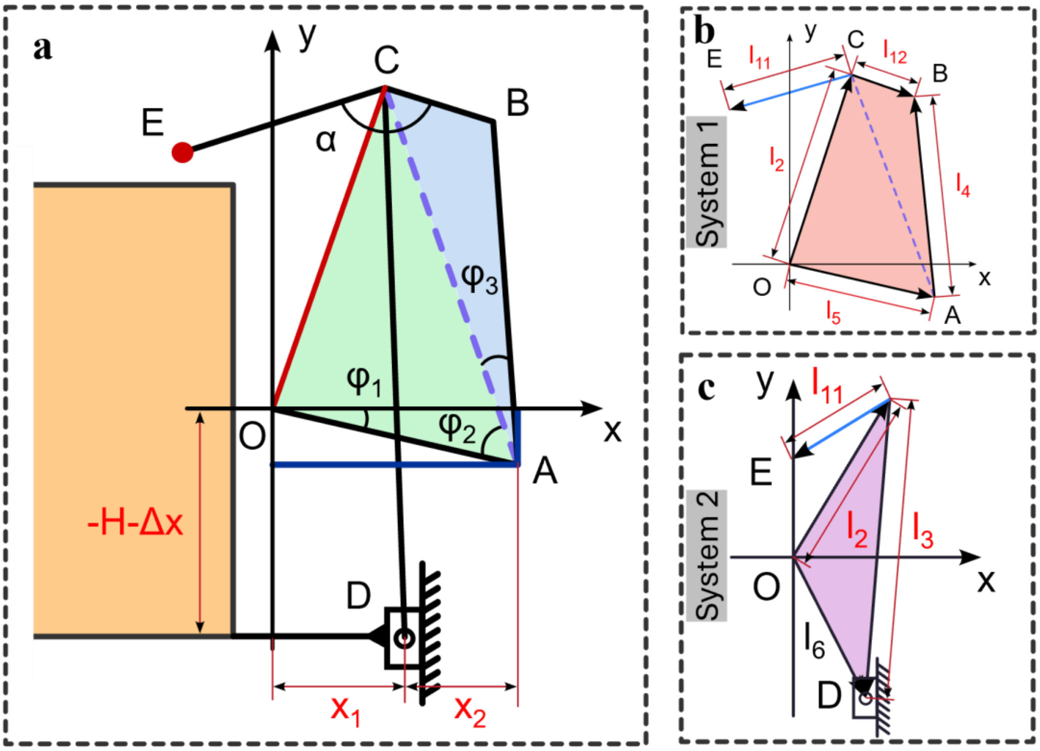

Figure. S13. Modeling analysis of the gripper switching frame. (a) Simplified overall model of the gripper. (b) Geometry of the first vector loop. (c) Simplified geometry of the second vector loop.

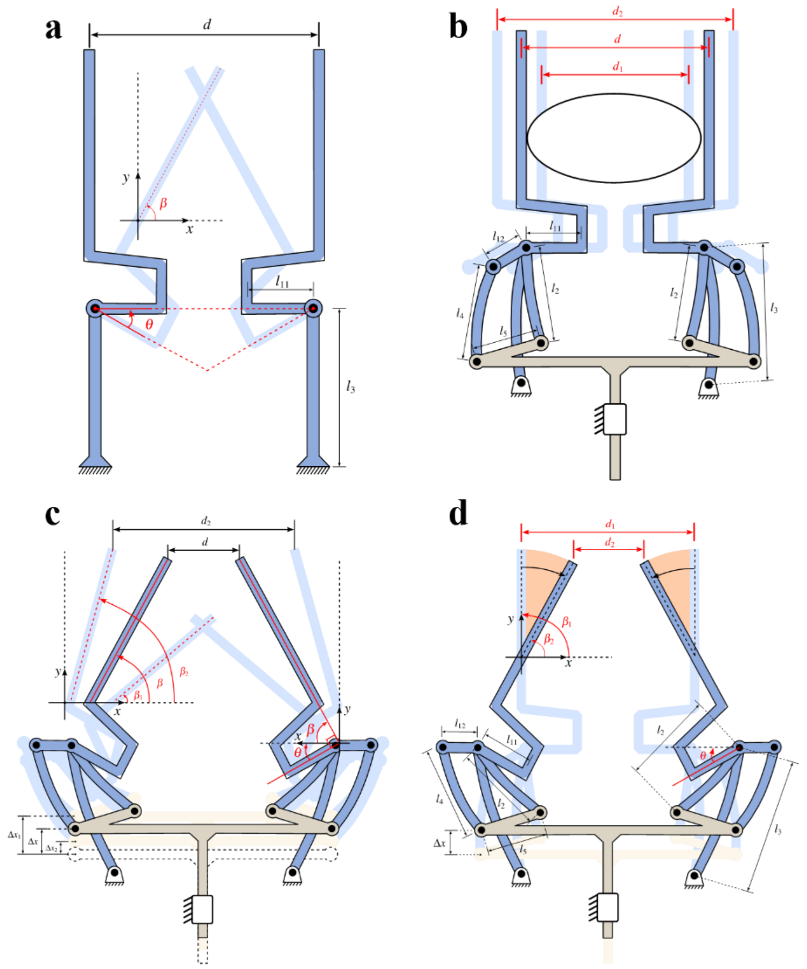

Figure. S14. Four distinct gripper configurations. (a) Under rotation about a fixed point, the target switching angle is met but interference occurs at the fingertip. (b) In the parallel state, the tip spacing is either too large or too small. (c) Over a very small upward stroke, the rotation angle is too small or too large. (d) A configuration that satisfies the grasping requirements of both modes.

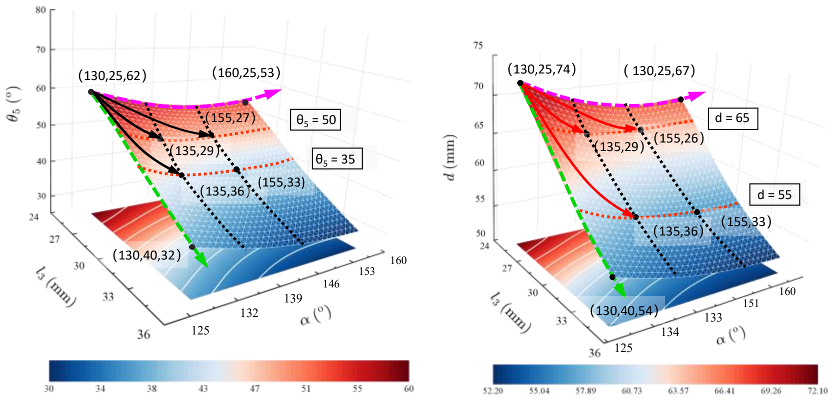

Figure. S15. Surface responses of the finger rotation angle and the change in fingertip spacing as functions of expansion length and expansion angle.

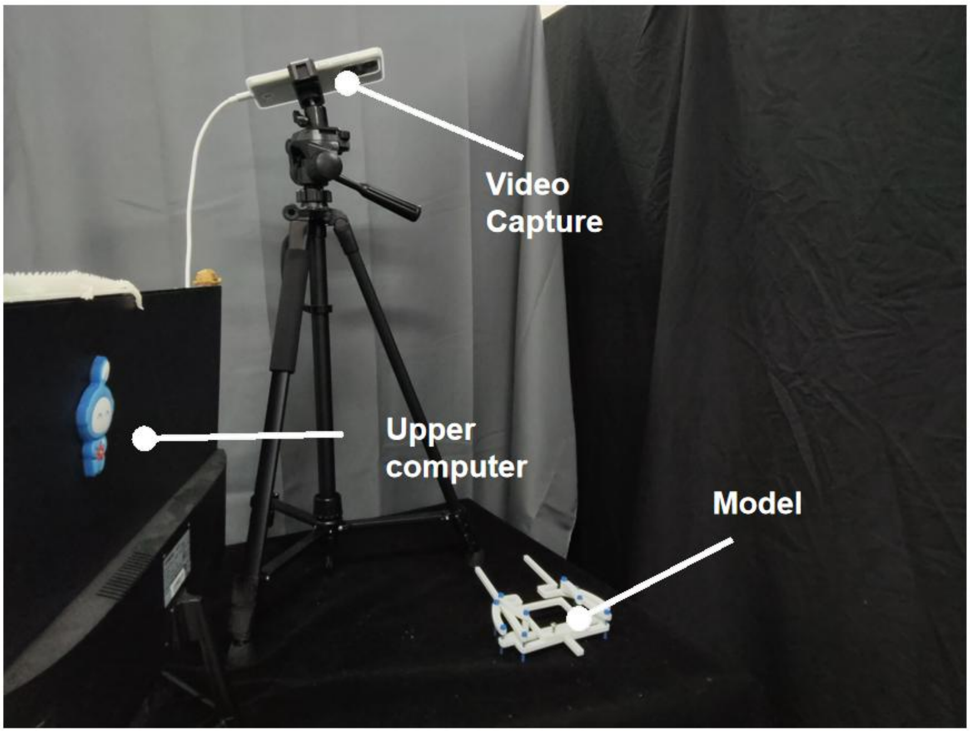

Figure. S16. Simplified model of the gripper system and its test and data acquisition setup.

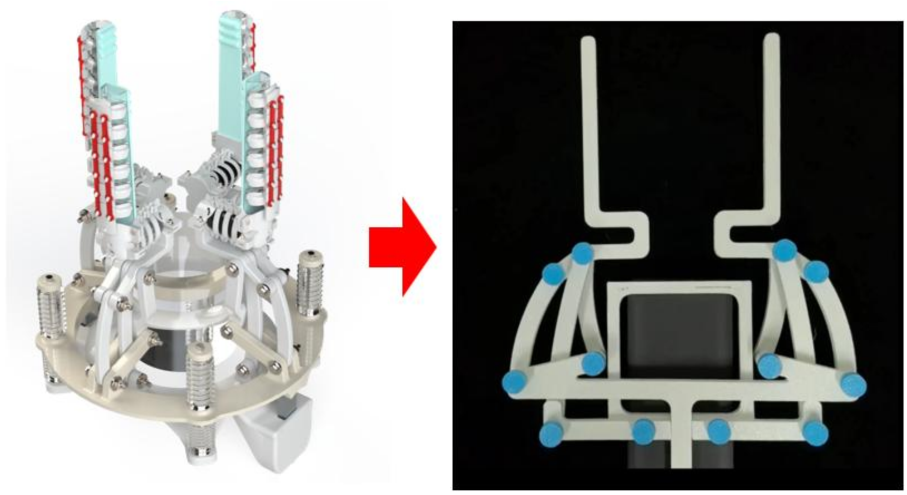

Figure. S17. Simplified structural model of the switching frame.

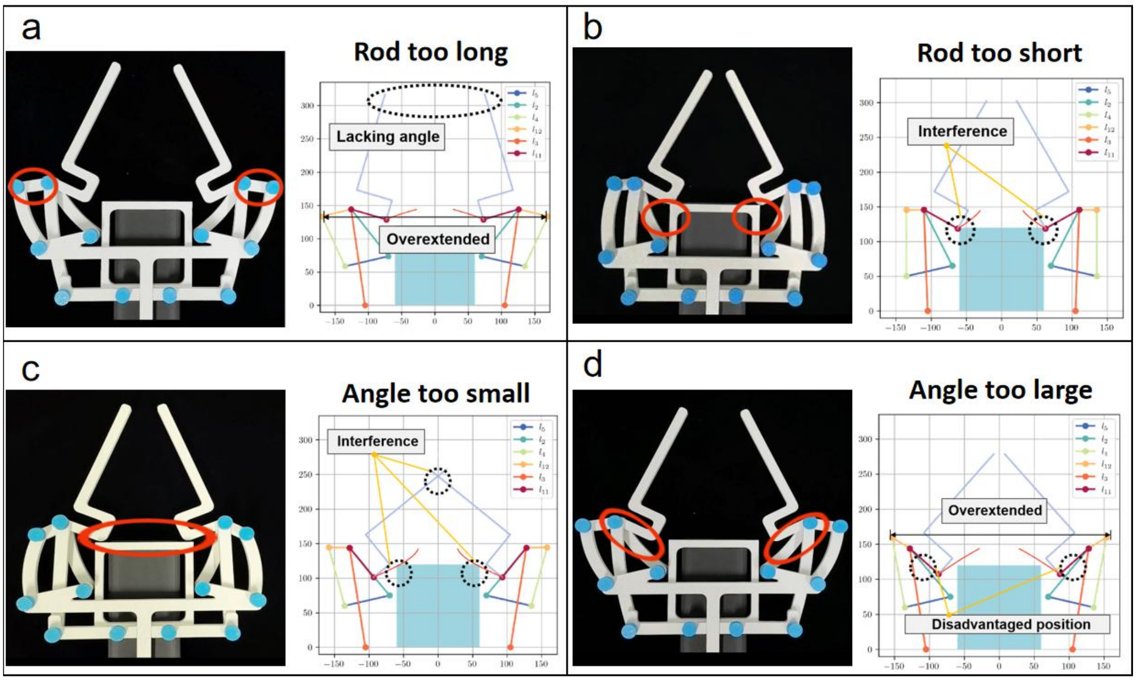

Figure. S18. Effect of design parameters on gripper motion. (a) An overlong link overhang length produces an excessively large tip spacing for the switching frame. (b) A short overhang length causes interference between the soft finger and the palm region. (c) An overhang angle that is too small leads to interference during switching. (d) An overhang angle that is too large yields excessive tip spacing in the caged configuration and increases the likelihood of a dead point.

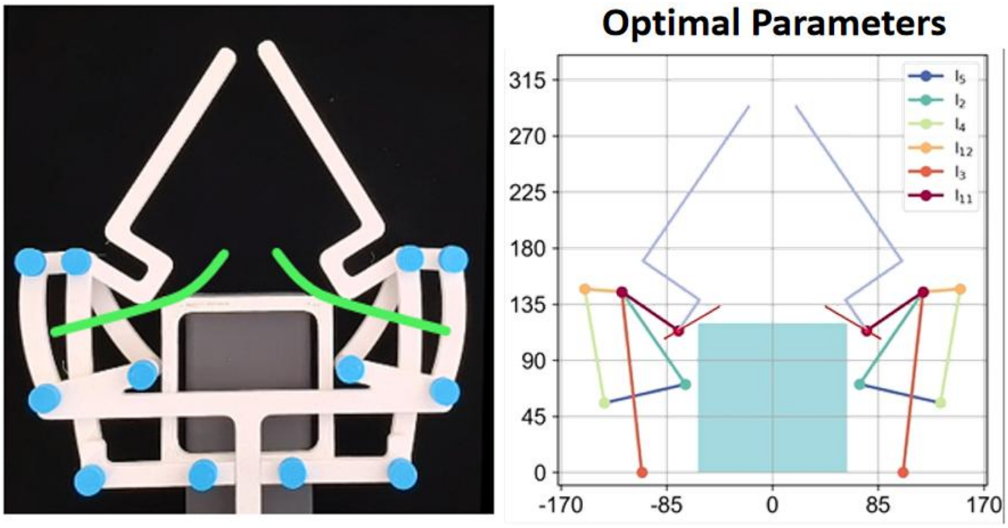

Figure. S19. Suitable design parameters of the switching frame.

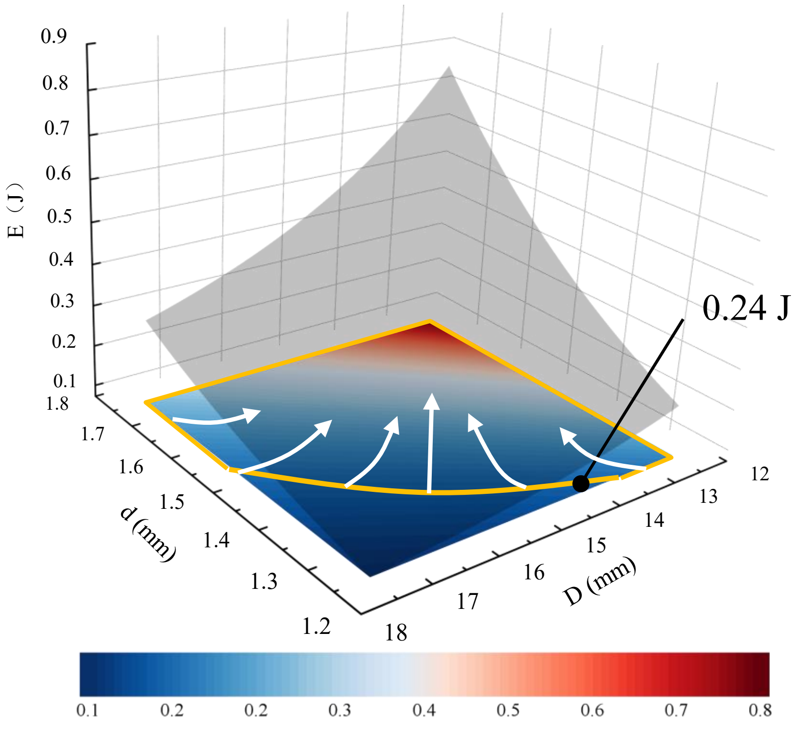

Figure. S20. Elastic potential energy of the spring.

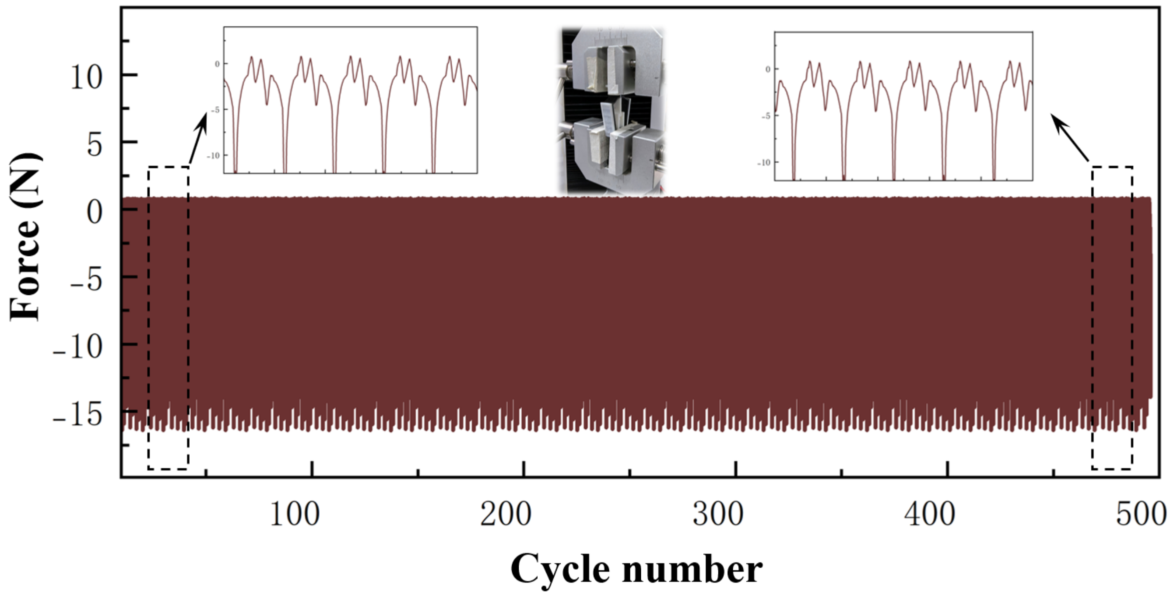

Figure.S21 Test curve of unit cell

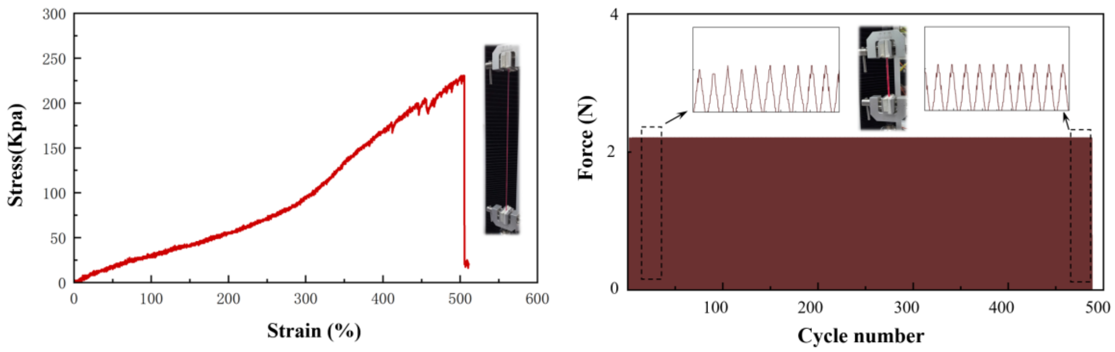

Figure.S22 Test curve of the elastic tendon

Table S1 Material parameters

Silicone type	C_{10}	C_{20}	C_{30}	$D_1/D_2/D_3$
Mold star 30	0.59101	0.28782	0.00013	0
Dragon skin 30	0.14084	-0.01435	0.00240	0
Ecoflex 00-30	0.01363	0.00019	0.00005	0

Table S2. Simulation parameters.

Symbol	Description	Value
m_a	Mass of ball	50g
m_b	Mass of the tentacle	40g
v_{a0}	Initial velocity of ball	1.5m/s
V_{b0}	Initial velocity of the center of mass of the tentacle	0
H	Ball hits the tentacle horizontally with a height	118mm
θ_0	Initial deflection angle of the tentacle	0°
e	The restitution coefficient of Newton's hypothesis	0.86
r_b	Total length of the tentacle	150mm
E_v	The elastic modulus of the elastic layer	From 30 to 90 Mpa
L_v	Second moment of area of the elastic layer on the ventral side of the tentacle	$192mm^4$
c	Damping coefficient of the polyurethane rubber	15N*s/rad
E_d	The elastic modulus of the limiting blocks	From 1.5 to 3Gpa
I_d	Second moment of area of the limiting blocks on the dorsal side of the tentacle	$1.4*10^5$ mm^4
